# P2Y_6_ Receptor Potentiates Pro-Inflammatory Responses in Macrophages and Exhibits Differential Roles in Atherosclerotic Lesion Development

**DOI:** 10.1371/journal.pone.0111385

**Published:** 2014-10-31

**Authors:** Ricardo A. Garcia, Mujing Yan, Debra Search, Rongan Zhang, Nancy L. Carson, Carol S. Ryan, Constance Smith-Monroy, Joanna Zheng, Jian Chen, Yan Kong, Huaping Tang, Samuel E. Hellings, Judith Wardwell-Swanson, Joseph E. Dinchuk, George C. Psaltis, David A. Gordon, Peter W. Glunz, Peter S. Gargalovic

**Affiliations:** 1 Cardiovascular Drug Discovery, Bristol-Myers Squibb Company, Pennington, New Jersey, United States of America; 2 Applied Genomics, Bristol-Myers Squibb Company, Pennington, New Jersey, United States of America; 3 Pharmaceutical Compound Optimization: Discovery Toxicology, Bristol-Myers Squibb Company, Pennington, New Jersey, United States of America; 4 Lead Evaluation, Bristol-Myers Squibb Company, Lawrenceville, New Jersey, United States of America; 5 Veterinary Sciences, Bristol-Myers Squibb Company, Pennington, New Jersey, United States of America; 6 Discovery Chemistry, Bristol-Myers Squibb Company, Pennington, New Jersey, United States of America; University of Padua, Italy

## Abstract

**Background:**

P2Y_6_, a purinergic receptor for UDP, is enriched in atherosclerotic lesions and is implicated in pro-inflammatory responses of key vascular cell types and macrophages. Evidence for its involvement in atherogenesis, however, has been lacking. Here we use cell-based studies and three murine models of atherogenesis to evaluate the impact of P2Y_6_ deficiency on atherosclerosis.

**Methodology/Principal Findings:**

Cell-based studies in 1321N1 astrocytoma cells, which lack functional P2Y_6_ receptors, showed that exogenous expression of P2Y_6_ induces a robust, receptor- and agonist-dependent secretion of inflammatory mediators IL-8, IL-6, MCP-1 and GRO1. P2Y_6_-mediated inflammatory responses were also observed, albeit to a lesser extent, in macrophages endogenously expressing P2Y_6_ and in acute peritonitis models of inflammation. To evaluate the role of P2Y_6_ in atherosclerotic lesion development, we used P2Y_6_-deficient mice in three mouse models of atherosclerosis. A 43% reduction in aortic arch plaque was observed in high fat-fed LDLR knockout mice lacking P2Y_6_ receptors in bone marrow-derived cells. In contrast, no effect on lesion development was observed in fat-fed whole body P2Y_6_xLDLR double knockout mice. Interestingly, in a model of enhanced vascular inflammation using angiotensin II, P2Y_6_ deficiency enhanced formation of aneurysms and exhibited a trend towards increased atherosclerosis in the aorta of LDLR knockout mice.

**Conclusions:**

P2Y_6_ receptor augments pro-inflammatory responses in macrophages and exhibits a pro-atherogenic role in hematopoietic cells. However, the overall impact of whole body P2Y_6_ deficiency on atherosclerosis appears to be modest and could reflect additional roles of P2Y_6_ in vascular disease pathophysiologies, such as aneurysm formation.

## Introduction

Mechanisms mediating inflammatory responses to tissue injury associated with atherosclerotic lesion development are critical to disease progression and clinical outcome. Recruitment of macrophages to atherosclerotic plaque and their activation is crucial in this process and depends on multiple pathways and stages of plaque development. During early stages of lesion formation, in so called “fatty streaks”, the inflammatory response is primarily driven by the interaction of oxidized lipids with vascular endothelial cells (ECs). On the other hand, in advanced plaque containing necrotic cores and cellular debris, activation of lesional macrophages is likely to play a significant role [1].

Nucleotides represent key signaling molecules with diverse biological actions. They can be released to the extracellular space under a variety of stress conditions, such as tissue damage, infection, mechanical stimulation and hypoxia, and are considered to be danger signals or part of damage-associated molecular patterns (DAMPs) [2]. Nucleotides contribute to the inflammatory response by interacting with purinergic receptors on the cellular surface, thus activating downstream signaling pathways. Indeed, purinergic P2Y (G-protein coupled receptors) and P2X (ligand-gated ion channels) receptors modulate a variety of immune pathologies and pathways associated with inflammation and immune cell activation [3]. Recent studies described the presence of P2Y receptors in murine atherosclerotic lesions and directly implicated P2Y_6_ in atherosclerosis [4]. Specifically, P2Y_6_ receptor expression in lesions was enhanced with plaque development and was enriched primarily in macrophage foam cells. Consistent with this observation, a survey of P2Y_6_ expression across tissues and cell lines also showed enrichment of this receptor in macrophages [5]. P2Y_6_ is a Gq-coupled receptor, activated exclusively by the nucleotide UDP. UDP has been found to promote the release of pro-atherogenic inflammatory cytokines and chemokines in macrophages and several other cell types [6]–[9]. In addition to macrophages, P2Y_6_ is also expressed in vascular ECs and vascular smooth muscle cells (SMCs). In ECs, expression of P2Y_6_ is elevated when stimulated with tumor necrosis factor α (TNF-α) and P2Y_6_ knockout (KO) mice exhibit reduced systemic inflammatory responses to acute lipopolysacharide (LPS) challenge [10]. By contrast, in SMCs, P2Y_6_ can modulate vasoconstriction responses to UDP [11], [12].

To directly evaluate the proposed role of P2Y_6_ in atherosclerosis, we used P2Y_6_ KO mice and examined the impact of P2Y_6_ deficiency on lesion development in three different murine disease models. We confirm that P2Y_6_ promotes secretion of pro-inflammatory cytokines in macrophages and show using bone marrow transplant studies that P2Y_6_ deficiency in hematopoietic cells exerts a protective role on atherosclerotic lesion development in low density lipoprotein receptor (LDLR) knockout (KO) mice. We then examined the effect of whole-body deficiency on lesion formation in a standard dietary LDLR KO model and angiotensin II-infused pro-inflammatory model to reveal a potentially novel role for P2Y_6_ in aneurysm formation.

## Materials and Methods

### Reagents

All cell culture reagents, Blasticidin, Hank's balanced salt solution, Dulbecco's phosphate buffered saline, Fluo-4 AM and pluronic acid were purchased from Life Technologies. UDP was purchased from Sigma, 3-phenacyl UDP was from Tocris Bioscience. THP-1 cell line was purchased from ATCC. The 1321N1 astrocytoma cell line and all the other chemicals were purchased from Sigma Aldrich unless otherwise stated. All the reagents for RNA extraction, cDNA synthesis, PCR and probes were purchased from Applied Biosystems. Reagents for siRNA transfection were from Thermo Scientific. Multiplex cytokine assay kits were purchased from EMD Millipore. Monosodium Urate (MSU) was from Enzo Life Sciences.

### Animals and Diet

Animal studies were performed according to guidelines established by the American Association for Accreditation of Laboratory Animal Care and protocols were approved by the Bristol-Myers Squibb-Hopewell Animal Care and Use Committee. The P2Y_6_ knockout line was generated by homologous recombination using targeting vector generated with the Lamda KOS system [13]. Genomic clone pKOS17 containing the 3rd exon of the P2Y_6_ gene with the entire open reading was used to construct the targeting vector. A LacZ reporter/neomycin resistant cassette was inserted into the targeting vector to replace the P2Y_6_ coding exon. The targeting vector was electroporated into 129/SvEvBrd ES cells. G418 resistant ES cells were isolated and correctly targeted clones identified by Southern blot analysis. Targeted ES cells were microinjected into C57BL/6 albino blastocysts and germline transmission was achieved by breeding chimera with C57BL/6 mice. Genotyping was carried by Southern blot and PCR analysis. Four rounds of speed congenics were performed at the Jackson Laboratory to convert the P2Y_6_ knockout mice into 99.9% C57BL/6J background. Mice were mated with LDL receptor knockout mice (Jackson Laboratory, Bar Harbor, ME) to create heterozygous and homozygous P2Y_6_ mice on an LDL receptor-deficient background. All healthy male and female mice from internal breeding were used for the various studies to maximize use of the litters. Thus, male and female mice were used for distinct experiments without bias. All mice were fed standard rodent chow during the acclimation phase. For atherosclerosis studies, mice were fed Western diet (Research Diets, D12079B) containing 0.2% cholesterol and 20% milk fat for times dictated by each study design.

### Western Diet and AngII Accelerated Atherosclerosis

Dietary induced atherosclerosis studies were carried out with female mice fed Western diet (Research Diets, D12079B) for 12 weeks. At the end of the dietary phase, aortas were harvested for plaque analyses. Accelerated atherosclerosis studies were carried out with male mice infused with angiotensin II via subcutaneously implanted osmotic minipumps (Ang II infusion rate: 500 ng/kg/min). The day following pump implantation, mice were fed Western diet (Research Diets, D12079B) for 4 weeks and aortas and hearts were harvested for plaque analysis and incidence of abdominal aortic aneurysms.

### Bone Marrow Transplantation

Male LDLR KO mice (8–10 weeks old) were subjected to lethal irradiation (1000 rads) using a cesium source and reconstituted with bone marrow-derived stem cells from P2Y_6_ KO mice (1X10^7^ donor cells/mouse). Recipient mice were maintained under pathogen-free conditions on standard rodent chow for 4 four weeks and then switched to Western Diet (Research Diets, D12079B) for 16 weeks. After 16 weeks of Western diet, aortas and hearts were harvested for plaque analysis.

### Atherosclerosis Analysis

Aortas were pinned to black wax plates and stained with Oil Red O stock solution (1.8% v/v). Stained aortas were immersed in phosphate buffered saline (PBS) and photographed using a Nikon Digital Camera (Nikon Digital Sight DS-Fi1) mounted on Nikon Dissecting Microscope (SMZ 1000, Micron-Optics, Cedar Knolls, NJ). Image analysis was performed by NIS-Elements: Basic Research Version 3.0 (Micron-Optics, Cedar Knolls, NJ). Oil Red O stained lesion areas were measured and expressed as a percentage of total aorta area as described previously. To characterize plaque composition, histological analysis of lesions in the aortic root was performed via the Paigen method. Serial sections were stained with trichrome for lesion area measurements. Macrophage content was evaluated by CD68-positive staining via immunohistochemistry. A rat anti-mouse CD68 antibody (Cat. #: MCA1957; AbD Serotec, Raleigh, NC) was applied to paraffin-embedded and sectioned samples. Samples were processed as described [14].

### Abdominal Aortic Aneurysm Analysis

The incidence and severity of abdominal aortic aneurysms were evaluated by post-hoc image analysis of gross abdominal aortic specimens. Histological analysis of abdominal aortas (intra-renal portion) was performed following paraffin embedding. Sections were cut to a thickness of 6 µm and stained using Masson's Trichrome. Plaque composition, medial band integrity, adventitial structure and inflammation were evaluated.

### Peritonitis

Peritonitis studies were performed with 8–10 week old mice on standard rodent chow. Mice were injected with 4% thioglycollate solution to induce peritonitis. Mice were euthanized at designated times post injection (4-days to collect peritoneal macrophages). The abdominal cavity was lavaged with 10 ml of cold PBS to collect peritoneal macrophages. Approximately 100 µl of each cell suspension was analyzed for total cell count via automated cell counting and cell type using modified Wright Giemsa staining and light microscope evaluation. Remaining cell suspensions were used for cellular studies.

For experiments using monosodium urate crystals, crystals were prepared in PBS (pH 7.4) at a concentration of 2 mg/ml and injected at 1 mg/mouse intraperitoneally. 3P-UDP was prepared in PBS (pH 7.4) at concentration 2 mg/ml and injected at 1 mg/mouse. For co-administration, 0.5 ml of each compound was drawn up into one syringe and a total of 1 ml injected into each mouse. Following the injection, animals were placed back into their respective cages. For blood collection, mice were anesthetized with isofluorane and 0.5 ml blood taken into EDTA tubes via retro-orbital puncture. Blood was centrifuged (10,000 rpm, 10 min at 4°C) and plasma samples used for cytokine analysis. For evaluation of cell infiltration into the peritoneal cavity, mice were euthanized at 0–4 days post injection with MSU and cells harvested via intraperitoneal lavage using 10 ml of PBS. Lavage solution was stored on ice and cells counted (macrophages, neutrophils, lymphocytes, and mast cells). Differentiation of each cell type was carried out by chemical staining of lavage smears and identification by cell morphology via light microscopy according to standard procedures.

### Plasma Lipids Analysis

EDTA-anti-coagulated blood samples were taken by retro-orbital bleeding following a 4 hr fast and plasma was isolated by centrifugation. Plasma total cholesterol and triglycerides were analyzed enzymatically using a Siemens Advia 1800 automated chemistry analyzer (Siemens Healthcare Diagnostics).

### Foam Cell Assay

Freshly isolated mouse peritoneal macrophages (MPMs) were plated in 384 well thin bottom imaging plates at a density of 20,000 cells per well. The next day, cells were washed three times with PBS to remove non-adherent cells and Dulbecco's modified eagle medium (DMEM) containing 0.1% fatty acid free BSA (Sigma) and the ACAT inhibitor (DUP128) or DMSO was added. The cells were incubated for 1 hr at 37°C before the addition of 100 µg/ml acetylated LDL (Ac-LDL) (Biomedical Technologies). Incubation was continued for an additional 20 hr. Cells were fixed with 3.7% formaldehyde and stained with 0.5 µg/ml Nile red (Invitrogen) and 5 µg/ml Hoechst 33342 (Molecular Probes) in 50% glycerol. All liquid handling steps were performed using a Biomek Fx liquid handling system equipped with a BioTek ELx405 plate washer. The plates were scanned on a Cellomics VTi arrayscan and neutral lipid droplets were quantitated using the Compartmental Analysis Bioapplication.

### Isolation of Primary Human Monocytes

Human primary monocytes were isolated by a double density method as described by Danciger et al. [15]. Briefly, buffy coat from 120 ml human blood was mixed with equal volume of Dulbecco's PBS with 1 mM EDTA and under layered with equal volume of Ficoll-Paque plus (GE Healthcare). Centrifugation at 400x g for 20 min at room temperature produced a dense white band above red blood cells containing peripheral blood mononuclear cells (PBMCs). The PBMC layer was harvested and washed twice with PBS with 1 mM EDTA to remove platelets. Washed PBMC layer cells were re-suspended in Iscove's modified dulbecco's medium (IMDM) from Invitrogen with 10% fetal bovine serum (FBS) and carefully topped onto 46% iso-osmotic Percoll (GE Healthcare) prepared with PBS and IMDM according to [15]. The one step discontinuous gradients were centrifuged at 550 x g for 30 min at room temperature. The monocyte band formed at the interface between the lower and upper layers was harvested and washed with DPBS at 4oC. Freshly isolated cells were evaluated under microscope after Turks staining and Wrights stain (Richard-Allan Scientific). A typical preparation yields a monocyte purity of 80% or higher.

### Cell Culture

Mouse peritoneal macrophages were isolated 4-days after intra-peritoneal injection of 4% thioglycollate as described above. Freshly isolated mouse peritoneal macrophages were plated into 96-well cell culture plates and cultured at 37°C, 5% CO2 in DMEM, containing 20% FBS and 1x Antibiotic-Antimycotic (Invitrogen) at density of 80,000/well for fluorescence imaging plate reader (FLIPR) assay or 100,000/well for cytokine secretion assay. Non-adherent cells were removed by washing twice with DPBS the following day. The adherent cell monolayer was maintained in serum-free DMEM until further experiments.

Freshly isolated human peripheral monocytes were seeded in non-tissue culture treated 96-well plate at density of 100,000/well and cultured at 37°C, 5% CO_2_ in Macrophage Serum-Free Medium (SFM) (Invitrogen) supplemented with 100 ng/mL recombinant M-CSF (Genscript). Non-adherent cells were removed the next day and adherent cells maintained at 37°C, 5% CO_2_ in the same medium for five days prior to analysis for cytokine secretion, also in Macrophage SFM.

THP-1 monocytes were cultured at 37°C, 5% CO_2_ in RPMI medium (Invitrogen) supplemented with 10% FBS, 2 mM L-glutamine, 1 mM sodium pyruvate, 25 mM HEPES and 55 µM 2-mercaptoethanol. Prior to each experiment, THP-1 monocytes were plated into 96-well tissue culture plate at density of 80,000/well and differentiated with 10 nM phorbol myristate acetate in culture medium for three days at 37°C, 5% CO_2_.

1321N1 astrocytoma cells were grown at 37°C, 5% CO_2_ in high glucose DMEM supplemented with 10% FBS and were stably transfected with 1.8 µg pEF-DEST51 expression vector encoding human P2Y_6_ cDNA using electroporation with Gene Pulser (Bio-Rad). Transfected cells were grown in presence of 10ug/ml blasticidin. 10 days after transfection, single cell colonies were expanded in 8 µg/ml blasticidin culture medium and screened by response to 3P-UDP in a calcium mobilization assay using FLIPR. P2Y_6_ mRNA levels and receptor function were also evaluated in selected cell clones by real time RT-PCR and cytokine secretion assay.

### FLIPR Assay

Cells were plated in 96-well (black wall and clear bottom) plates to reach 80% confluence. On the day of assay, the cell culture medium was removed and cells loaded with calcium dye by incubating in Hank's balanced salt solution containing 4 µg/ml Fluo-4 AM dye, 10 mM HEPES buffer, pH 7.4 and 2.5 mM probenicid for 45 min at 37°C in 5% CO_2_. The P2Y_6_ agonist UDP or 3P-UDP was added to cells using the FLIPR Tetra Cellular Screening System and calcium mobilization was recorded immediately.

### Real Time RT-PCR

Total RNA from cells was extracted on ABI 6100 Nucleic Acid PrepStation following manufacturer's protocol and processed by qScript reverse transcriptase (Quanta Biosciences) to obtain cDNA. P2Y_6_ genes were quantified by real time PCR in ABI7900 HT Sequence Detection System using SYBR Green with 500 nM primers: forward oligo GTGAGGATTTCAAGCGACTGC, reverse oligo TCCCCTCTGGCGTAGTTATAGA for mouse P2Y_6_; forward oligo CCACCACCTGTGTCTACCG, reverse oligo GCCAGAGCAAGGTTTAGGGT for human P2Y_6_. The expression of P2Y_6_ was normalized to a housekeeping gene, RPL30.

### Cytokine Secretion Assay

Cells cultured in 96-well plates were treated with indicated reagents in serum-free medium for indicated time points. Where not noted, macrophage incubations were usually carried out for 16 hr at 37°C in 5% CO_2_ cell culture media, harvested and analyte concentrations evaluated by multiplex ELISA using Milliplex assay kits and Luminex 200 system according to manufacturer's instructions.

### Evaluation of UDP and 3P-UDP Stability

UDP and 3P-UDP were added to 96-well plate wells in cell culture medium in presence or absence of THP-1 cells. Medium samples were collected after 0, 5 hr and 20 hr of incubation. Remaining UDP and 3P-UDP in the culture medium was quantified by liquid chromatography with tandem mass spectrometry (LC-MS/MS)-based bioanalytical method developed for this purpose. Briefly, the UHPLC Nexera system (ultra high pressure LC) from Shimadzu (Columbia, MD) was used, which consists of two pumps (LC-30AD), a column oven (CTO-30A), and a SIL-30AC autosampler with Rack Changer II that maintained samples at 10°C during analysis. The analytical column used was an Acquity BEH C18 2.1 mm x 50 mm, 1.7 µm (Waters Corporation, Milford, MA) with column oven set at 40°C. The mobile phase consisted of 20 mM ammonium acetate (NH4Ac), 0.15% dibutylamine (DBA) in water (A) and methanol (B), and was delivered at a flow rate of 0.6 mL/min. The analytes were eluted chromatographically with gradient as following: 0–0.3 min, 5%B; 0.7 min, 50%B; 1.6 min, 90%B; and 1.7 to 4 min, 5%B as equilibrium. The retention times for UDP and 3P-UDP and IS were 1.12, 1.26 and 1.31 min, respectively with above gradient. The total analysis time was 1.8 min. The UHPLC was interfaced to an API 4000 QTRAP tandem mass spectrometer (Applied Biosystems, Concord, Ontario, Canada) equipped with a turbo ionspray interface operating in the positive ionization mode. Ultra-high-purity (UHP) nitrogen was used as the nebulizing and heating gases at settings of 35 for nebulization and 45 for heater. The source temperature was set at 600°C. Detection of each analyte was achieved through selected reaction monitoring (SRM). Ions representing the precursor (M+H) + species for UDP and 3P-UDP and IS were selected in quadrupole 1 and collisionally dissociated with UHP nitrogen at a setting of 6 to generate specific product ions, which were subsequently monitored by quadrupole 3. The SRM transitions monitored for UDP, 3P-UDP and IS were 403/79, 521/159 and 560/79 respectively.

UDP and 3P-UDP were also incubated with 1321N1_P2Y_6_ expression cell line in serum-free medium with or without 1 unit/ml apyrase (Sigma). After a 5 hr incubation period, the cell culture medium was collected and secreted cytokines/chemokines quantified by multiplex ELISA.

### P2Y_6_ Gene Knock Down by siRNA in THP-1 Cells

Human P2Y_6_ siRNA oligo CGCUGAACAUCUGUGUCAU and control non-target oligo (Accell siRNA, Invitrogen) were used to transfect THP-1 cells. On the day of transfection, THP-1 monocytes were spun down and re-suspended in Accell culture media containing 1 µM siRNA at density 0.8 million/ml. After a 24 hr incubation at 37oC in 5% CO2, equal volume of THP-1 growth medium containing 20 nM PMA was added and 200 µl of the mixture were seeded to a 96-well cell culture plate. Transfected THP-1 cells were differentiated in the 96-well plate at 37oC in 5% CO2 for three days before treatment with P2Y_6_ agonist.

### Data and Statistical Analysis

Data are reported as mean ± standard error of the mean. Statistical analyses were performed using a Student's unpaired t-test. For evaluation of aortic aneurysm incidence, Fisher's exact statistical significance test was used. Results were considered statistically significant at p<0.05.

## Results

### Secretion of Pro-atherogenic Inflammatory Cytokines in Human Astrocytoma Cells Expressing P2Y_6_

**Figure 1:**
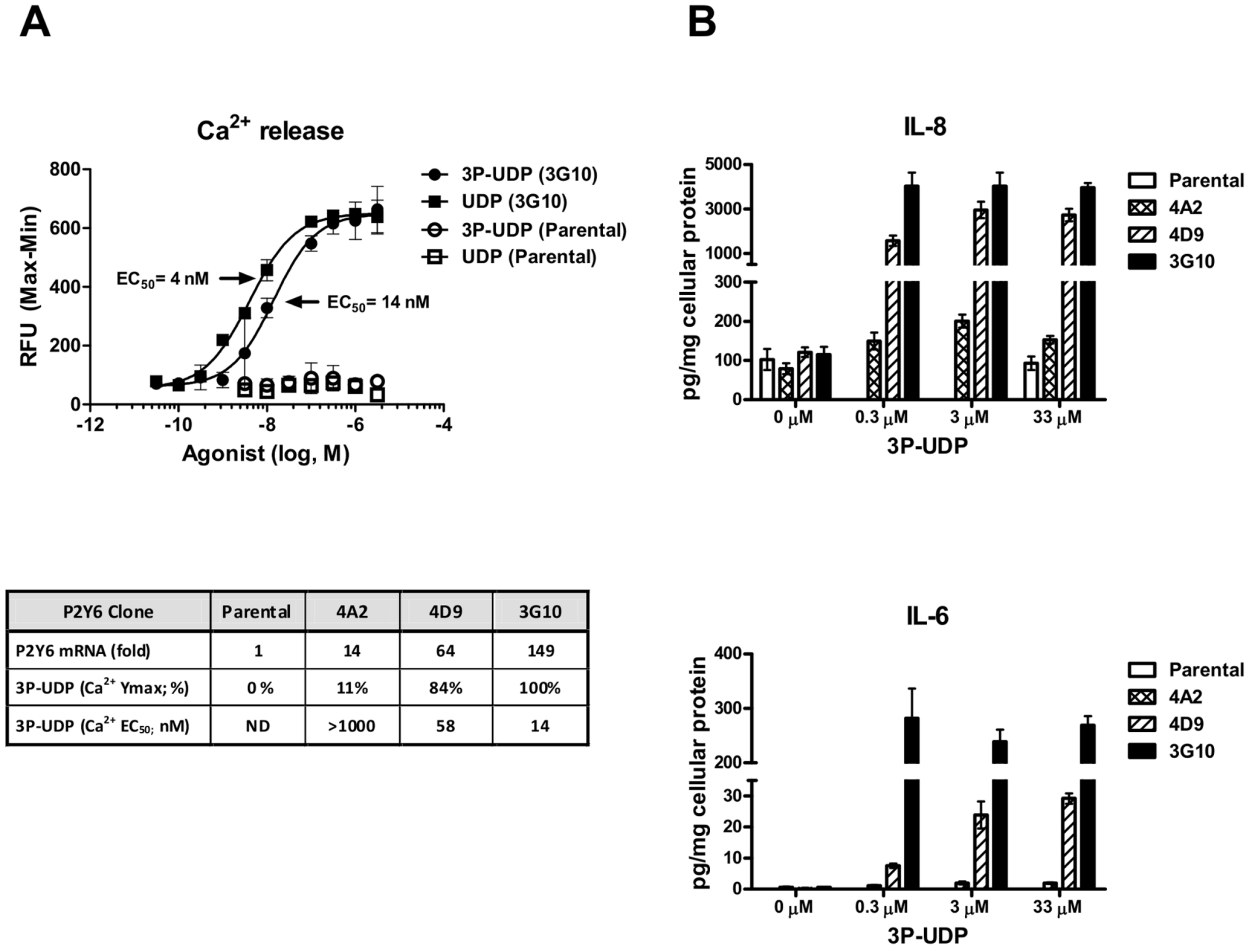
P2Y_6_ expression mediates release of calcium and pro-inflammatory cytokines in 1321N1 cells. Evaluation of calcium release and cytokine secretion to cell culture media in 1321N1 astrocytoma cells stably expressing human P2Y_6_ receptor. **A**) Isolated 1321N1 clones expressing varying levels of P2Y_6_ mRNA and mock transfected cells (Parental) were incubated with increasing concentrations of UDP and 3P-UDP. Calcium mobilization was measured to generate concentration response curves with examples shown for 3G10 clone and parental cell line. For each clone, achieved maximal calcium response is expressed as percentage relative to 3G10 clone (set as 100%) together with calculated EC_50_ values (ND  =  not determined) and relative P2Y_6_ mRNA levels (relative to parental cells, which had very low but detectable P2Y_6_ mRNA). **B**) 1321N1 clones were incubated for 20 hr with increasing concentrations of 3P-UDP and cytokines in cell culture medium quantified using multiplex ELISA as described in Materials and Methods. Each cytokine was normalized to cellular protein content. The data are presented as mean +/− SD of replicate wells.

1321N1 astrocytoma cells are widely utilized as cellular background to study P2Y receptor signaling predominantly due to absence of large fraction of P2Y receptor subtypes. Earlier work in these cells demonstrated release of interleukin 8 (IL-8) in response to UDP when P2Y_6_ was expressed [6]. To extend these findings, we generated a range of cell lines stably expressing human P2Y_6_ at varying levels and evaluated their responses to known P2Y_6_ agonists UDP and 3-phenacyl UDP (3P-UDP) [16]. Incubation of these cells with UDP and 3P-UDP increased intracellular calcium levels and led to release of IL-8 and IL-6 to the extracellular medium in a P2Y_6_-dependent manner (**Figure 1A, 1B**). The observed response was directly correlated with P2Y_6_ expression level across the stable clones and was lost in parental cells or cells treated with medium pre-incubated with a nucleotide-degrading enzyme apyrase (**Figure S1**). Indeed, UDP is susceptible to degradation by extracellular nucleotidases, which limits its use in prolonged experiments (**Figure S1**). Because 3P-UDP has similar potency (Figure 1A; 14 nM vs. 4 nM), but increased stability and receptor selectivity, it was used in the majority of our studies. Further analysis of cell supernatants revealed that P2Y_6_ agonism induced secretion of a number of additional cytokines, including monocyte chemotactic protein 1 (MCP-1) and growth factor induced 1 (GRO1), all of which have been linked to atherogenesis (**Figure 1** **and Figure S1**) [17]–[19].

### P2Y_6_ Modulates Inflammatory Cytokine Secretion in THP-1 and Primary Macrophages

**Figure 2:**
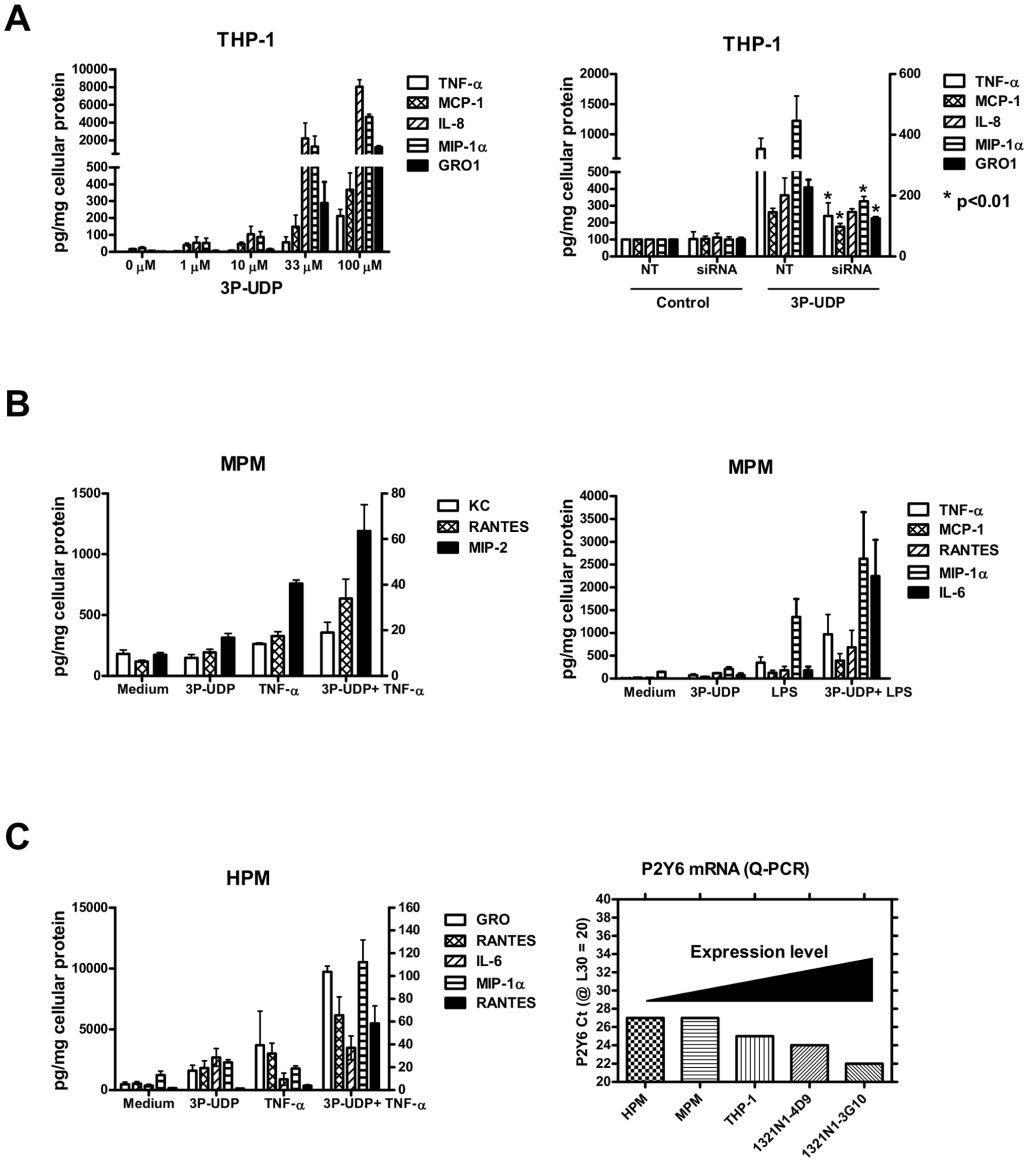
P2Y_6_ receptor agonism modulates inflammatory cytokine release from macrophages. Activation of P2Y_6_ with 3P-UDP potentiates secretion of pro-inflammatory cytokines in macrophages. **A**) Differentiated THP-1 macrophages were incubated for 16 hr with increasing concentrations of 3P-UDP and cytokines in cell medium analyzed using multiplex ELISA. Cytokines which were affected by 3P-UDP treatment are shown (left graph). On the right, THP-1 monocytes were differentiated in presence of control non-targeting (NT) or P2Y_6_ siRNA (siRNA) and treated with 33 µM 3P-UDP for 16 hr to determine cytokine levels in medium. The level of P2Y_6_ mRNA knockdown was 78%. The data are presented as mean +/−SD of replicate wells from a representative experiment. Significance P2Y_6_ siRNA vs non-targeting siRNA: *p<0.01. **B**) Adhered mouse peritoneal macrophages were incubated in serum-free medium for 16 hr with combinations of 100 µM 3P-UDP either alone or in the presence of additional inflammatory stimulus (0.1 ng/mL TNF-α or 10 ng/mL LPS) and analyzed for cytokines levels in cultured medium using ELISA. **C**) Human primary monocyte-derived macrophages were incubated with 100 µM 3P-UDP either alone or in the presence of 10 pg/mL TNF-α and analyzed for cytokine levels as described in Materials and Methods. Relative expression of P2Y_6_ mRNA in each cell line was compared using quantitative PCR Threshold Cycle (Ct) values. P2Y_6_ Ct values were normalized to RPL30 (set at Ct  =  20) and plotted to illustrate relative expression compared to abundant RPL30 housekeeping gene (Ct  =  20) or genes expressed at the very low levels (Ct> 35).

To evaluate the role of P2Y_6_ in macrophage cytokine responses, we incubated THP-1 cells with increasing concentrations of 3P-UDP and assayed 16 cytokines in the cell culture medium. Consistent with data observed in 1321N1 cells, we found several inflammatory cytokines to be secreted from THP-1 cells in a concentration-dependent manner. Furthermore, the response was observed to be significantly diminished when expression of P2Y_6_ was selectively inhibited via siRNA treatment (**Figure 2A** **and Figure S2**). Interestingly, the cytokine response to 3P-UDP was much less pronounced in MPMs. Incubation with 3P-UDP, however, enhanced cytokine responses to other pro-inflammatory stimuli such as LPS and TNFα (**Figure 2B**). A similar response was observed in human primary blood monocyte-derived macrophages (HPMs). The observed differences in responsiveness could be due to the variation in P2Y_6_ expression since HPMs and MPMs express relatively lower levels of P2Y_6_ than THP-1 cells (∼ 4-fold) and much lower than 1321N1-P2Y6 clones (∼ 32-fold less when compared to 3G10 clone) (**Figure 2C**).

### P2Y_6_ KO Mice Exhibit Normal Gross Phenotype with Marginally Diminished Response to Inflammatory Challenge

**Figure 3:**
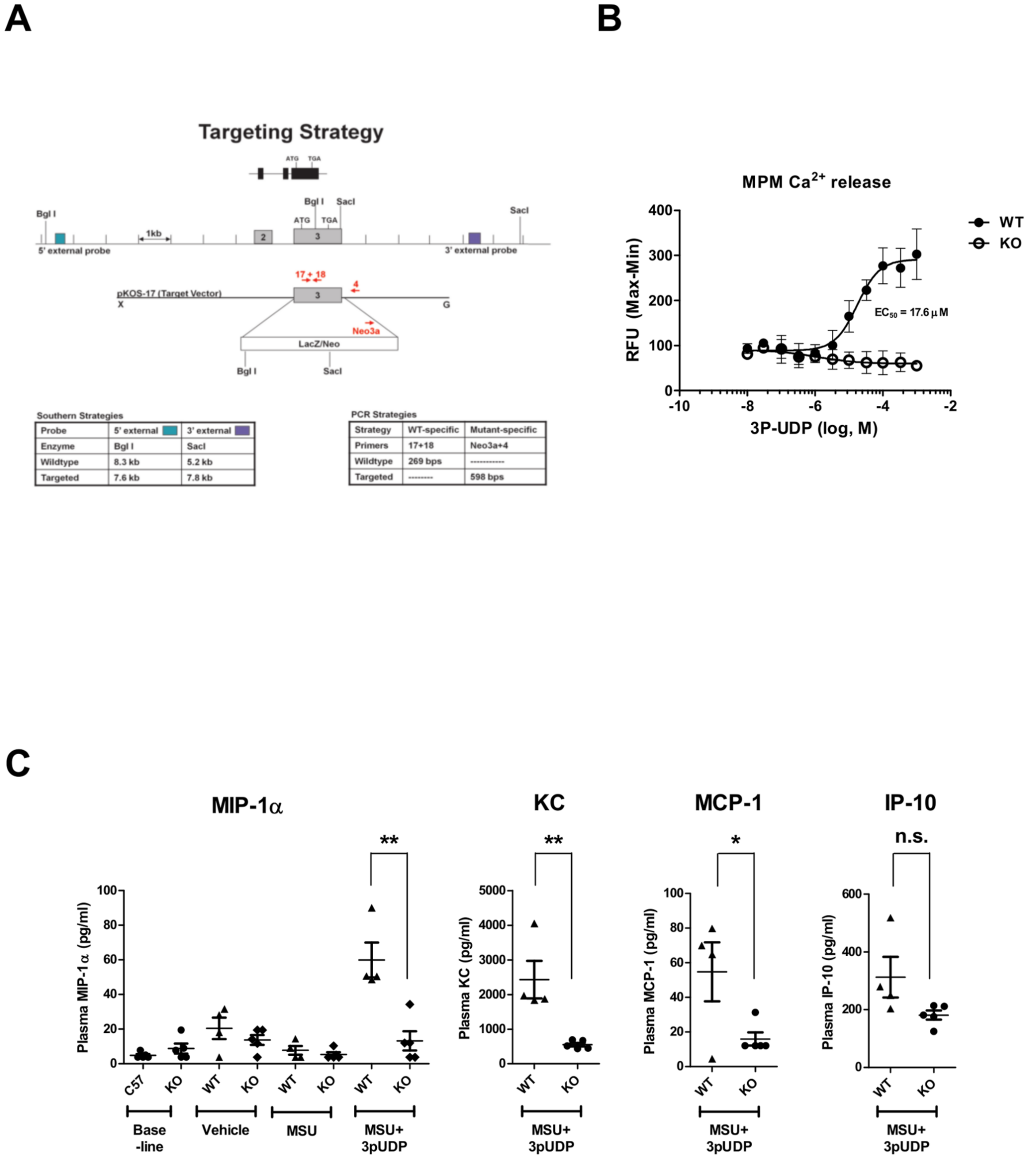
P2Y_6_ knockout mice exhibit reduced responsiveness to inflammatory challenge. Generation and evaluation of P2Y_6_ KO mice. **A**) Targeting strategy used to replace the 3^rd^ exon of the P2Y_6_ gene containing the entire open reading frame with a pKOS-17 vector containing β-galactosidase (LacZ) reporter and neomycin resistance (Neo) cassette. The second and third exon of the P2Y_6_ gene are depicted as grey boxes with indicated translational start and stop codons. Location of Southern Blot probes is shown in green and blue, and PCR genotyping primers are shown in red. **B**) MPMs from P2Y_6_ knockout mice (KO) and wild type littermates (WT) were incubated with increasing concentrations of 3P-UDP to generate calcium concentration response curves. Calculated EC_50_ value for WT MPMs is shown. **C**) P2Y_6_ KO males and WT male littermates (n =  4–5 per group) were subjected to intra-peritoneal challenge with vehicle, MSU or combination of MSU and 3P-UDP (1mg/mouse each) as described in Materials and Methods. Blood was collected 1 hr after injection and cytokines in plasma measured by multiplex ELISA. Cytokines that displayed differential responses in KO mice are shown. Baseline (unchallenged mice) and vehicle treated mice are also depicted for MIP-1α. Significance KO vs WT: **p<0.01 *p<0.05. *Not significant, n.s.*

To directly examine the role of P2Y_6_ in atherosclerotic lesion development, we generated P2Y_6_ KO mice by disrupting the third exon, which contains the entire open reading frame (**Figure 3A**). Functional P2Y_6_ deficiency was confirmed by the absence of calcium release to 3P-UDP agonism in MPMs isolated from P2Y_6_ KO mice (**Figure 3B**).

As reported previously, P2Y_6_ deficient mice exhibited no overt phenotype and were born with expected Mendelian pattern [11]. Additional extensive phenotyping of KO mice also did not reveal any significant perturbations when compared to wild type (WT) littermates with the exception of diminished responsiveness to inflammatory challenges (**Figure S3**). MSU crystals are known to stimulate inflammatory responses that cause gout via a mechanism that appears to involve the P2Y_6_ receptor [20]. We used acute intra-peritoneal challenge with a threshold dose of MSU (1 mg/mouse), which elicited minimal elevation in plasma cytokines (**Figure 3C**). Co-administration of 3P-UDP with MSU elevated the concentration of inflammatory cytokines in plasma and this response was diminished in P2Y_6_ KO mice, as evident by significantly reduced levels of macrophage inflammatory protein 1α (MIP-1α), chemokine C-X-C motif ligand 1(KC) and monocyte chemoattractant protein 1 (MCP-1) (**Figure 3C**). Although not significant, there was also a trend toward decreased IFN gamma-inducible protein 10 (IP-10) levels. Attenuated responsiveness was also observed in MPMs isolated from P2Y_6_ KO mice when challenged with 3P-UDP and MSU or TNF-α in vitro, although effects were not as robust as seen in vivo (**Figure S4**). This is not surprising, since other non-macrophage cell types expressing P2Y6 are likely to contribute to overall inflammatory responses to P2Y_6_ activation in vivo, which is consistent with previous observations made in endothelial cells [10]. These findings suggest that the P2Y_6_ receptor modulates inflammatory pathways in vitro and in vivo but its activation alone is insufficient to drive robust inflammatory responses. Instead, P2Y_6_ appears to function by potentiating responses to other pro-inflammatory agents.

### P2Y_6_ Deficiency has Regional and Model-dependent Effects on Murine Atherosclerotic Lesion Development

**Figure 4:**
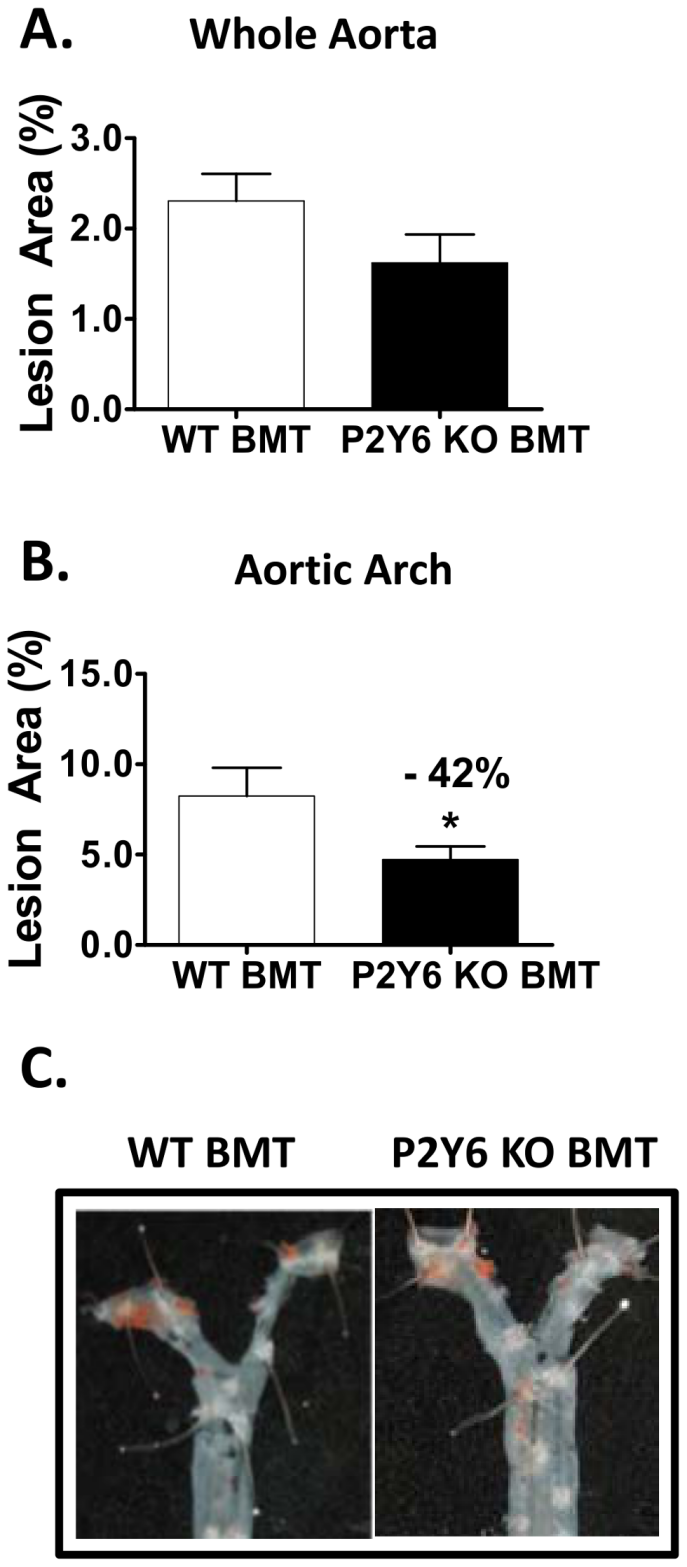
Atherosclerosis development with P2Y_6_-deficient bone marrow reconstitution. **A**) Quantification of en face lesion area with oil red O staining in the whole aorta from the ascending aorta to the iliac bifurcation and **B**) aortic arch. **C**) Representative images of aortic arch atherosclerosis. Group sizes: n = 9 P2Y_6_ WT transplanted mice; n = 14 P2Y_6_ KO transplanted mice. Significance vs. control: *p<0.05. *Not significant, n.s.*

To study the effects of leukocyte-derived P2Y_6_ on atherosclerosis, the bone marrow transplantation (BMT) model was used [21]. Bone marrow-derived stem cells were harvested from P2Y_6_ KO mice (on a C57BL/6 background) and P2Y_6_ wild type littermate controls and transplanted into whole-body irradiated male LDLR KO recipient mice. Following a 4 week engraftment and recovery phase, all mice were switched to Western diet for 16 weeks. As shown in **Figure 4A**, en face analysis of atherosclerosis in the whole aorta did not reveal significant differences between P2Y_6_ KO or P2Y_6_ WT transplanted mice. Although not significant, a modest trend towards reduced en face staining was noted with P2Y_6_ KO transplantation (30% vs. WT). In the aortic arch, plaque area was significantly reduced (42%) with P2Y_6_ KO transplantation vs. control (p<0.05; **Figure 4B-C**). The observed decrease in lesion formation in this model is consistent with our macrophage studies indicating a pro-inflammatory and pro-atherogenic role of P2Y_6_ in leukocytes. No changes in plasma cholesterol or triglycerides were observed (**Figure S5**). This lack of impact on plasma lipid levels is consistent with P2Y_6_ modulating primarily inflammatory and not lipid-mediated signaling pathways.

**Figure 5:**
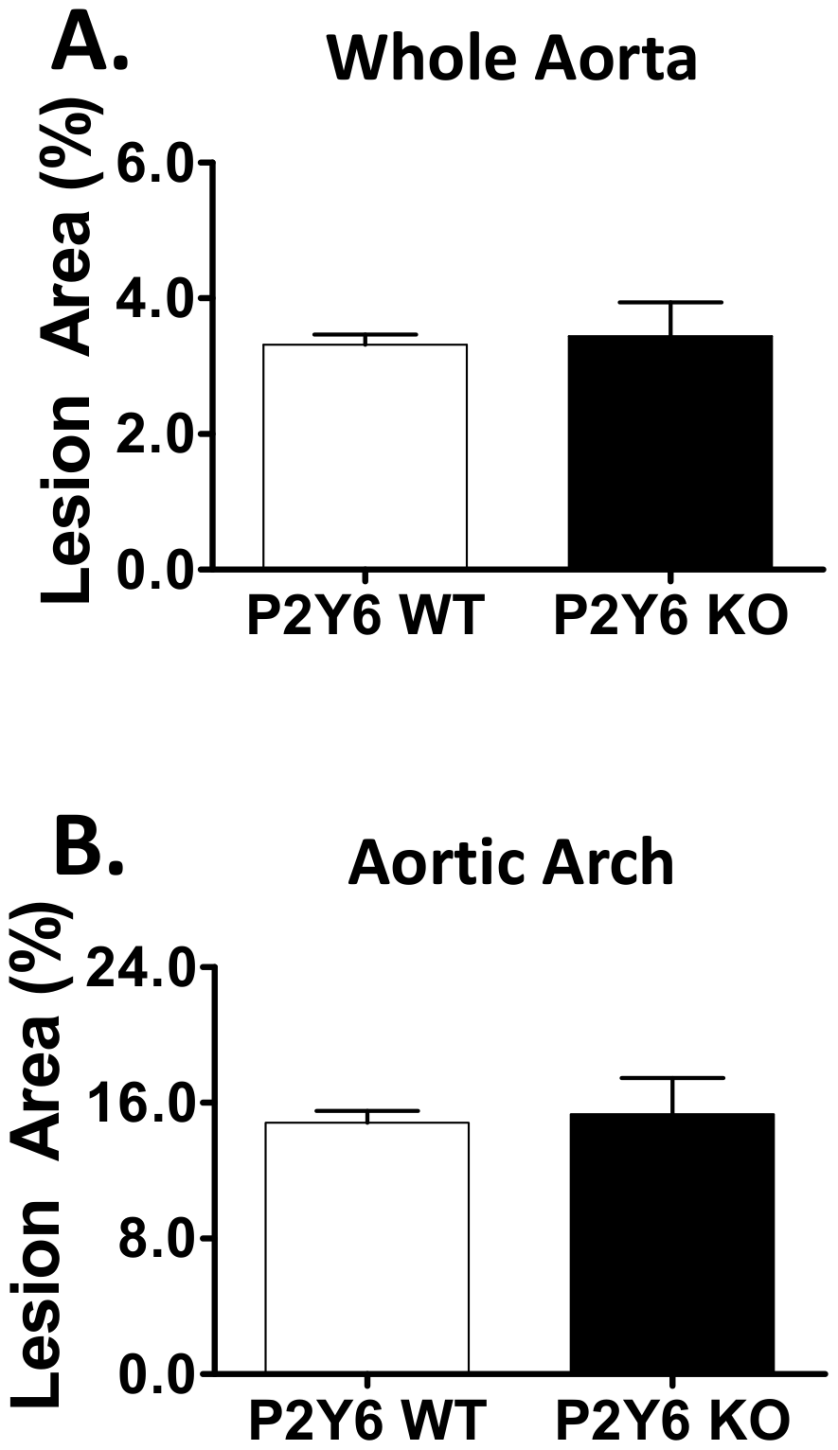
Atherosclerosis development with Western Diet Feeding. En face plaque quantification of excised mouse aortas using oil red O. **A**) Lesion area measured in the whole aorta from the ascending aorta to the iliac bifurcation. **B**) Regional atherosclerosis in the aortic arch. The percentage of positive stained area with respect to total analyzed aortic area. Group sizes: n = 12 P2Y_6_ WT mice; n = 13 P2Y_6_ KO mice.

The impact of whole-body P2Y_6_ receptor deficiency on diet-induced atherosclerotic lesion development was evaluated using female LDLR KO mice. Female P2Y_6_ KO x LDLR KO mice (hereafter referred to as “P2Y_6_ KO”) and P2Y_6_ wild type littermates (P2Y_6_ WT x LDLR KO or “P2Y_6_ WT”) were placed on a pro-atherogenic Western diet for 12 weeks. Mice gained weight normally over the dietary feeding phase, plasma lipids were similar between groups (**Figure S5**) and no aberrations in their basic physiology or behavior were detected. Plaque development in the aorta of mice was measured at the end of the feeding phase. All mice developed fatty streak lesions in their aortas as indicated by analysis of en face oil red O staining (**Figure 5**). As shown in **Figure 5A**, en face analysis of the whole aorta revealed similar levels of oil red O-stained plaques between P2Y_6_ KO and P2Y_6_ WT mice. Plaque development in the aortic arch was also similar between P2Y_6_ KO and P2Y_6_ WT mice (**Figure 5B**). These data indicate a lack of effect of whole body deficiency on lesion formation in this model.

**Figure 6:**
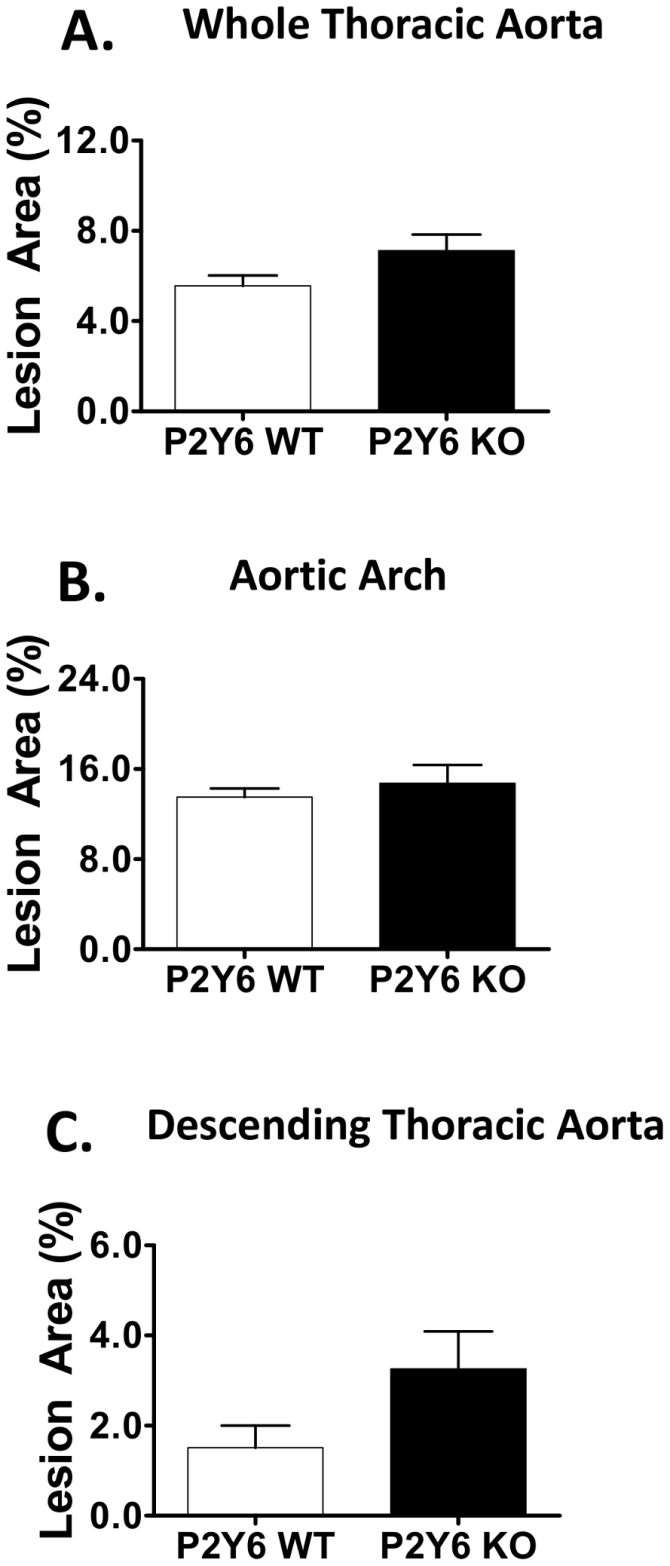
Accelerated Atherosclerosis development with Angiotensin II infusion. Atherosclerosis development measured in the aorta with oil red O. **A**) Quantification of en face lesion area in the thoracic aorta (ascending aorta to the diaphragm). Regional atherosclerosis in **B**) aortic arch and **C**) descending thoracic aorta segments. Group sizes: n = 9 P2Y_6_ WT mice; n = 14 P2Y_6_ KO mice. The fold change in P2Y_6_ lesion area is given relative to P2Y_6_ wild type levels. *Not significant, n.s.*

Given these findings, we wanted to further assess the impact of P2Y_6_ on atherogenesis in the context of enhanced vascular inflammation and subjected P2Y_6_ KO or WT control mice (both on an LDLR KO background) to angiotensin II infusion in addition to dietary challenge. Studies in LDLR and apolipoprotein E KO (ApoE KO) mice have shown that chronic infusion of angiotensin II promotes accelerated atherosclerosis development, vascular inflammation and extensive abdominal aneurysm formation [22], [23]. After 4 weeks of treatment with angiotensin II and high-fat diet, en face plaque analysis of the whole thoracic aorta, aortic arch or descending thoracic aorta did not reveal significant differences between P2Y_6_ KO and P2Y_6_ WT mice (**Figure 6A-C**). A non-significant trend towards increased plaque development was noted in the descending thoracic aorta with P2Y_6_ deficiency (2.2 fold increased vs. control, p = 0.07; **Figure 6C**). Unlike the BMT and pro-atherogenic dietary experiments described above, a modest increase in plasma total cholesterol was observed with P2Y_6_ deficiency vs. control (21%, p<0.01; **Figure S5**).

**Figure 7:**
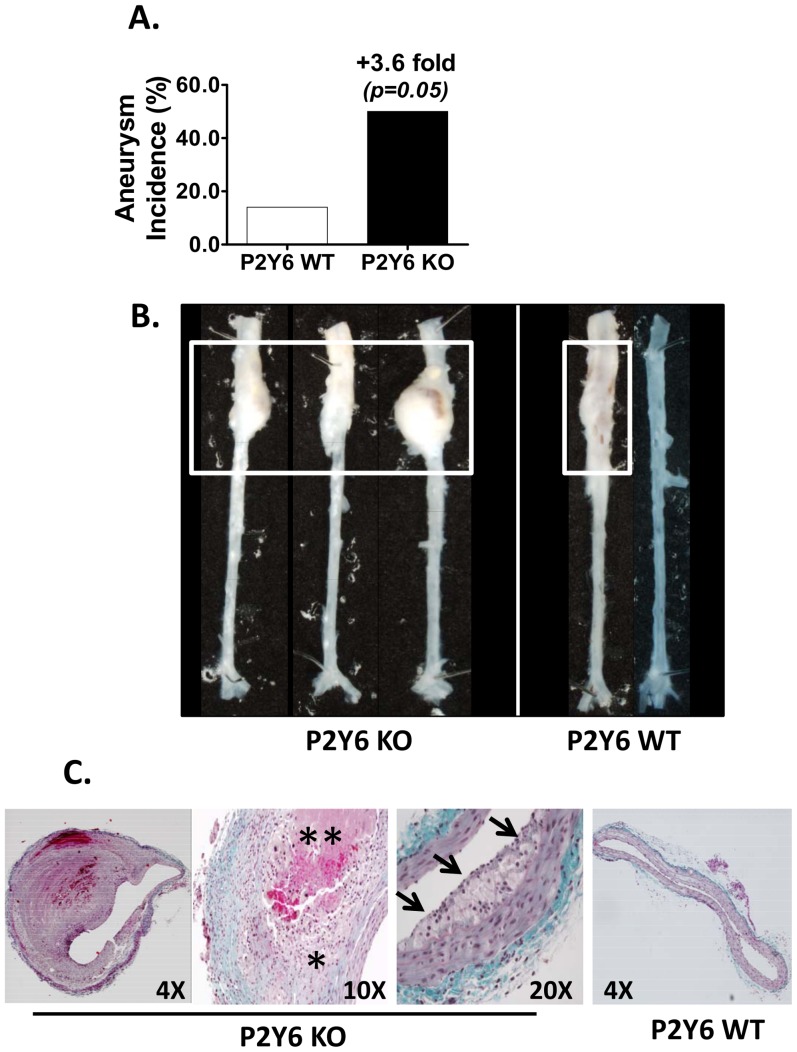
Abdominal Aortic Aneurysms. **A**) Incidence of aneurysm development in the abdominal aorta. **B**) Representative images of abdominal aortic aneurysms for P2Y_6_ KO mice and P2Y_6_ wild-type mice. Aneurysm-positive and -negative aortas are given for P2Y_6_ KO wild type mice. Aneurysms are demarcated by the white boxes. **C**) Trichrome-stained histological sections of aneurysms taken from the supra-renal section of the abdominal aorta. Sections were cut from the center of the aneurysm mass. The single asterisk denotes the highly inflamed and remodeled adventitial mass. The double asterisk denotes a region of apparent hemorrhage. Neo-intimal plaque is shown by the arrows. The image at the far right shows an abdominal aorta cross-section taken from P2Y_6_ wild type mice. The magnification scale for each image is shown.

Administration of angiotensin II is known to cause increased incidence of abdominal aortic aneurysms in mice and we evaluated aneurysm formation in both strains of mice via analysis of excised abdominal aortas. Interestingly, we found that with P2Y_6_ KOs, aneurysm incidence increased 3.6 fold vs. P2Y_6_ WT control (**Figure 7A**). When aneurysms were present, they typically formed in the supra-renal segment of the abdominal aorta. Aneurysm presence was unambiguous and clearly visible to the naked eye. **Figure 7B** shows representative examples of aneurysms detected in the abdominal aortas of P2Y_6_ KO and P2Y_6_ WT mice. For comparisons, an aorta from a P2Y_6_ WT mouse that lacked aneurysms development is also shown (**Figure 7B**, rightmost image). To further evaluate the aneurysms, cross-sectional examinations of aneurysms were carried out following trichrome-staining of histological sections. **Figure 7C** shows a large eccentric aneurysm mass on an abdominal aorta section from the P2Y_6_ KO group analyzed at 4X magnification. We noted extensive inflammatory cell infiltration and vascular hemorrhage, as illustrated by the asterisks within the remodeled adventitia (10X magnification). Extensive matrix remodeling is evident both within the adventitia and also within the neointima. Neo-intimal hyperplasia/plaque development is highlighted by the arrows at the luminal surface of the vessel (20X magnification). For comparison, a histological section from a P2Y_6_ WT littermate lacking an aneurysm is also shown (**Figure 7C**, rightmost image at 4X magnification). Despite the observed propensity of P2Y_6_ KO mice to form aneurysms, this histological examination did not reveal any morphological or structural differences in aneurysms derived from P2Y_6_ KO vs. P2Y_6_ WT controls.

## Discussion

In this study we generated P2Y_6_ knockout mice, and for the first time, directly examined the role of the P2Y_6_ receptor in the development of atherosclerosis. Consistent with a proposed pro-inflammatory and pro-atherogenic role for this receptor in leukocytes, we found significant and region-specific reductions in atherosclerotic plaque formation in mice lacking P2Y_6_ in bone marrow-derived cells. This finding was also corroborated in primary macrophages in vitro, where P2Y_6_ activation augmented secretion of pro-inflammatory mediators, including MCP-1 and IL-8, which are known to play important roles in atherosclerotic lesion formation [24], [25].

Our finding that P2Y_6_ signaling leads to inflammatory activation of macrophages is consistent with other reports [6]–[8], [11]. Interestingly however, our data suggest that the magnitude of this inflammatory response is highly dependent on the level of receptor expression. For example, in 3G10, 4D9 and THP-1 cell lines, which expressed the highest levels of P2Y_6_ mRNA, incubation with agonist alone was sufficient to elicit the inflammatory response. By contrast, P2Y_6_ agonism failed to induce cytokine secretion in primary peritoneal macrophages, which expressed approximately 32-fold less P2Y_6_ mRNA than 3G10 cells. Primary macrophages displayed pro-inflammatory responses only in the presence of additional inflammatory stimulus, such as LPS, TNF-α or MSU **(** **Figure 2** **and Figure S4).** Similar observations in peritoneal macrophages were noted by Bar et al. [11], although in their studies UDP was used, which we found to be unstable with prolonged incubations in cell-containing medium (**Figure S1**). Importantly, results from our BMT studies indicate that P2Y_6_ modulation of pro-inflammatory signaling in macrophages in vitro is highly relevant to the pathophysiology of atherosclerosis and helps explain the impact on disease development in vivo, where multiple inflammatory mediators likely coexist in the milieu of the atherosclerotic plaque. Macrophage recruitment and associated P2Y_6_ expression are also increased during lesion development in ApoE KO mice, perhaps reflecting an increase in macrophage content as well as enhanced regional inflammatory status associated with atherogenesis [4]. In our studies, deficiency of P2Y_6_ in macrophages showed the largest impact on lesion formation in the aortic arch, an area which also had the highest lesion burden and macrophage content in this model.

P2Y_6_ mRNA is not restricted to leukocytes. P2Y_6_ expression has also been detected in vascular SMCs and ECs, which could also influence lesion development [4], [10], [11]. In fact, recent studies in human coronary ECs have shown that P2Y_6_ is induced with TNF-α treatment and P2Y_6_ promotes inflammatory gene induction in ECs in vitro and in vivo [10]. To extend our work in macrophages and further assess the impact of P2Y_6_ deficiency in other cell types, we used the LDLR KO double knockout mouse model. We selected this model over the ApoE KO since ApoE is known to modulate macrophage inflammatory responses and is directly involved in the regulation of lipid trafficking [26]. In contrast to bone marrow deficiency, whole body P2Y_6_ deletion failed to impact lesion formation in this model. This finding was surprising and indicates potentially complex and differential roles for the P2Y_6_ receptor in vascular disease development. It is unlikely that these differential responses are due to effects on lipid or cholesterol metabolism pathways given the lack of effect on plasma lipids between P2Y_6_ KO and P2Y_6_ WT mice (**Figure S5**). Moreover, when we evaluated P2Y_6_ effects on macrophage lipoprotein uptake, we found that MPMs from P2Y_6_ KO mice did not differ from WT MPMs (**Figure S6**).

In light of the protective role of P2Y_6_ deficiency in leukocytes, as indicated by our BMT studies, we investigated the role of P2Y_6_ in vascular inflammation using the angiotensin II-accelerated atherosclerosis model. Administration of angiotensin II leads to robust enhancements of vascular inflammation via activation of NF-kB pathways, resulting in increased expression of chemokines and adhesion molecules in the vascular wall. The consequence is enhanced inflammatory cell recruitment, tissue injury and structural remodeling of the arterial medial layer due to smooth muscle cell activation, proliferation and changes in composition of extracellular matrix. Vascular inflammation and associated remodeling leads to accelerated lipid deposition and intimal hyperplasia in the vessel wall [22], [27]. In addition to these effects, weakening of the arterial wall and an increased propensity for abdominal aortic aneurysm formation are also caused by angiotensin II infusion in these pro-atherogenic mouse models [22], [23].

Although our studies focused on the involvement of P2Y_6_ in pro-atherogenic macrophage function, aneurysm formation is thought to primarily involve medial SMCs [28], [29]. P2Y_6_ is highly expressed in lesional SMCs, but its exact role in these cells is unclear [11]. Published studies suggest that P2Y_6_ can modulate vascular tone and enhance proliferation and migration of SMCs in vitro [11], [12], [30]. Despite the predicted anti-inflammatory effect of P2Y_6_ KO, our findings in the angiotensin II-infused LDLR KO model were similar to results obtained in the Western diet fed model, with P2Y_6_ deficiency resulting in little impact on lesion development in the whole thoracic aorta and the aortic arch (compare **Fig. 5** **and** **6**). Surprisingly, we observed an increase in aneurysm event rates in angiotensin II-infused P2Y_6_ KO mice. These findings suggest that differential and perhaps opposing roles for this receptor exist in macrophages versus other vascular cell types. Because of the high expression of P2Y_6_ in vascular SMCs and the predominant role of these cells in aneurysm development, it is plausible that the observed susceptibility to form aneurysms could be related to a vital role of P2Y_6_ in maintaining SMC function. Thus, the atheroprotective effects of P2Y_6_ deficiency in macrophages could be negated in the setting of pro-aneurysm development, where P2Y_6_-regulated smooth muscle cell function is considerably more important. Future studies are warranted to characterize the physiological roles of P2Y_6_ in vascular SMCs, specifically as it relates to vascular tissue remodeling and aneurysm formation.

We cannot exclude the possibility that other unexplored factors might have some impact on these findings. For example, it is possible that in the LDLR KO model, which is primarily driven by dietary cholesterol elevation, P2Y_6_ modulation of vascular inflammatory pathways might not be as prevalent as in other disease models. Moreover, in the BMT model, lethal irradiation of hyperlipidemic LDLR KO mice is known to significantly influence localization and composition of atherosclerotic lesions and could contribute to differential responses observed in our studies [31].

In conclusion, we used P2Y_6_ KO mice to examine the role of this receptor in atherogenesis. We found significant pro-inflammatory and pro-atherogenic roles of this receptor in macrophages. By contrast, we found P2Y_6_ deficiency to be associated with increased susceptibility to aneurysm formation, suggesting a differential functional role of this receptor in vascular cells. Thus, P2Y_6_ activity may also play a favorable role in maintaining the structure and function of the arterial wall.

## Supporting Information

Figure S1 **P2Y_6_ Agonist-Stimulated Release of Cytokines in Cell Lines and Agonist Stability.** **A**) 1321N1_P2Y_6_ stable cell line (clone 3G10) was incubated with UDP and 3P-UDP (33 µM) for 5 hours in the presence or absence of 1 unit/ml apyrase. After incubation, IL-8 in culture medium was measured by ELISA. **B**) THP-1 macrophages were incubated with UDP and 3P-UDP (33 µM) for indicated times and the level of UDP and 3P-UDP in culture medium quantified by liquid chromatography and mass spectrometry as described in Materials and Methods. The same experiment was carried out also in the absence of cells. Data were expressed as % remaining relative to 0 hr time point. **C**) 1321N1 clones were incubated for 20 hrs with increasing concentrations of 3P-UDP and cytokines in cell culture medium quantified using multiplex ELISA as described in Figure 1 and Materials and Methods. Each cytokine was normalized to cellular protein content. The data are presented as mean +/− SD of replicate wells.
(PDF)

Figure S2 **P2Y Receptors in Macrophages.** Relative expression of P2Y receptors in mouse primary peritoneal macrophages (**A**) and THP-1 cells before and after differentiation (**B**) was analyzed by quantitative PCR. The Threshold Cycle (Ct) value for each P2Y was normalized to RPL30 (set at Ct =  20) and plotted to illustrate relative abundance when compared to RPL30 housekeeping gene (Ct  =  20) or genes expressed at the very low levels (Ct> 35). **C**) P2Y_6_ siRNA treatment of THP-1 macrophages described in **Figure 2A** selectively reduced P2Y_6_ expression level (78% when compared to non-targeted oligonucleotide) and did not lead to reduced expression of other measured P2Y receptors (P2Y_1_, P2Y_2_ and P2Y_11_).
(PDF)

Figure S3 **Phenotypic Characterization of P2Y_6_ KO Mice and WT Littermate Controls.** Immunology and Cardiovascular/Metabolism studies were carried out with mice ranging in age from 12 to 25 weeks. In most cases, male mice were used. Results from studies using female mice are underlined. With few exceptions, most assays did not reveal differences between strains. *Note: the increase in bone density detected in this assay by DEXA scan was not observed in a subsequent cohort of mice by micro computed tomography imaging.
(PDF)

Figure S4 **Attenuated Responsiveness of MPMs Isolated from P2Y_6_ KO Mice Post 3P-UDP and MSU or TNF-α Challenge.** Thioglycollate-elicited MPMs were isolated from P2Y_6_ KO mice and incubated for 16 hrs with **A**) 3P-UDP (33 µM) +/− MSU (200 µg/ml) or **B**) 3P-UDP (33 µM) +/− TNF-α (0.1 ng/ml). Cytokines in cell culture medium were analyzed using multiplex ELISA. Results are presented as mean +/− SD from replicate wells. Similar results were obtained in multiple experiments using different animals. Only cytokines which displayed trend or were statistically significantly different between KO and WT are shown. Significance KO vs. WT: *p<0.05.
(PDF)

Figure S5 **Plasma Cholesterol and Triglycerides.** Total plasma cholesterol and triglycerides from P2Y_6_ WT and P2Y_6_ KO mice. Bone marrow transplantation study: **A**) Plasma total cholesterol and **B**) triglycerides in male mice (n = 9, P2Y_6_ WT BMT; n = 14, P2Y_6_ KO BMT). Western diet study: **C**) Plasma total cholesterol and **D**) triglycerides in female mice (n = 13, P2Y_6_ WT; n = 14, P2Y_6_ KO). Angiotensin II accelerated atherosclerosis study: **E**) Plasma total cholesterol and **F**) triglycerides in male mice (n = 14, P2Y_6_ WT; n = 12, P2Y_6_ KO). Significance KO vs. WT: *p<0.01. Values are mean ± SE.
(PDF)

Figure S6 **Acetylated LDL Uptake by MPMs.** MPMs isolated from P2Y_6_ KO mice, their WT littermates or age matched C57BL/6 mice were incubated for 20 hrs with 100 µg/ml of acetylated LDL (Biomedical Technologies) in the presence of 0.1% lipid-free BSA and the ACAT inhibitor DUP128 or DMSO. Cells were fixed and stained with Nile Red and Hoechst 33342. Neutral lipid content measured by high-content imaging and quantified by the Compartmental Analysis Bioapplication. Lipid accumulation was expressed as CircSpotTotalArea. The data are presented as mean +/−SD of replicate wells from a representative experiment. **A**) Quantification of cellular neutral lipid droplet area in DMSO vs. DUP128 (30 µM) treated MPMs from P2Y_6_ KO, WT and C57BL/6 mice. **B**) Concentration response curve for DUP128 in MPMs isolated from P2Y_6_ KO, WT and C57BL/6 mice.
(PDF)

## References

[1] Moore KJ, Tabas I (2011) Macrophages in the pathogenesis of atherosclerosis. Cell 145: 341–355. 21529710 10.1016/j.cell.2011.04.005 PMC3111065

[2] Rubartelli A, Lotze MT (2007) Inside, outside, upside down: damage-associated molecular-pattern molecules (DAMPs) and redox. Trends Immunol 28: 429–436. 17845865 10.1016/j.it.2007.08.004

[3] Junger WG (2011) Immune cell regulation by autocrine purinergic signalling. Nat Rev Immunol 11: 201–212. 21331080 10.1038/nri2938 PMC4209705

[4] Guns PJ, Hendrickx J, Van Assche T, Fransen P, Bult H (2010) P2Y receptors and atherosclerosis in apolipoprotein E-deficient mice. Br J Pharmacol 159: 326–336. 20050854 10.1111/j.1476-5381.2009.00497.x PMC2825354

[5] Lattin JE, Schroder K, Su AI, Walker JR, Zhang J, et al (2008) Expression analysis of G Protein-Coupled Receptors in mouse macrophages. Immunome Res 4: 5. 18442421 10.1186/1745-7580-4-5 PMC2394514

[6] Warny M, Aboudola S, Robson SC, Sevigny J, Communi D, et al (2001) P2Y(6) nucleotide receptor mediates monocyte interleukin-8 production in response to UDP or lipopolysaccharide. J Biol Chem 276: 26051–26056. 11349132 10.1074/jbc.M102568200

[7] Kukulski F, Ben Yebdri F, Lefebvre J, Warny M, Tessier PA, et al (2007) Extracellular nucleotides mediate LPS-induced neutrophil migration in vitro and in vivo. J Leukoc Biol 81: 1269–1275. 17322022 10.1189/jlb.1206758 PMC5239669

[8] Cox MA, Gomes B, Palmer K, Du K, Wiekowski M, et al (2005) The pyrimidinergic P2Y6 receptor mediates a novel release of proinflammatory cytokines and chemokines in monocytic cells stimulated with UDP. Biochem Biophys Res Commun 330: 467–473. 15796906 10.1016/j.bbrc.2005.03.004

[9] Kim B, Jeong HK, Kim JH, Lee SY, Jou I, et al (2011) Uridine 5'-diphosphate induces chemokine expression in microglia and astrocytes through activation of the P2Y6 receptor. J Immunol 186: 3701–3709. 21317391 10.4049/jimmunol.1000212

[10] Riegel AK, Faigle M, Zug S, Rosenberger P, Robaye B, et al (2011) Selective induction of endothelial P2Y6 nucleotide receptor promotes vascular inflammation. Blood 117: 2548–2555. 21173118 10.1182/blood-2010-10-313957 PMC3062416

[11] Bar I, Guns PJ, Metallo J, Cammarata D, Wilkin F, et al (2008) Knockout mice reveal a role for P2Y6 receptor in macrophages, endothelial cells, and vascular smooth muscle cells. Mol Pharmacol 74: 777–784. 18523137 10.1124/mol.108.046904

[12] Kauffenstein G, Drouin A, Thorin-Trescases N, Bachelard H, Robaye B, et al (2010) NTPDase1 (CD39) controls nucleotide-dependent vasoconstriction in mouse. Cardiovasc Res 85: 204–213. 19640930 10.1093/cvr/cvp265 PMC3694873

[13] Wattler S, Kelly M, Nehls M (1999) Construction of gene targeting vectors from lambda KOS genomic libraries. BioTechniques 26: 1150–1156, 1158, 1160. 10376154 10.2144/99266rr02

[14] Garcia RA, Search DJ, Lupisella JA, Ostrowski J, Guan B, et al (2013) 11beta-hydroxysteroid dehydrogenase type 1 gene knockout attenuates atherosclerosis and in vivo foam cell formation in hyperlipidemic apoE(−)/(−) mice. PloS one 8: e53192. 23383297 10.1371/journal.pone.0053192 PMC3562192

[15] Seager Danciger J, Lutz M, Hama S, Cruz D, Castrillo A, et al (2004) Method for large scale isolation, culture and cryopreservation of human monocytes suitable for chemotaxis, cellular adhesion assays, macrophage and dendritic cell differentiation. J Immunol Methods 288: 123–134. 15183091 10.1016/j.jim.2004.03.003

[16] El-Tayeb A, Qi A, Muller CE (2006) Synthesis and structure-activity relationships of uracil nucleotide derivatives and analogues as agonists at human P2Y2, P2Y4, and P2Y6 receptors. J Med Chem 49: 7076–7087. 17125260 10.1021/jm060848j

[17] Schwartz D, Andalibi A, Chaverri-Almada L, Berliner JA, Kirchgessner T, et al (1994) Role of the GRO family of chemokines in monocyte adhesion to MM-LDL-stimulated endothelium. The Journal of clinical investigation 94: 1968–1973. 7962543 10.1172/JCI117548 PMC294616

[18] Boring L, Gosling J, Cleary M, Charo IF (1998) Decreased lesion formation in CCR2−/− mice reveals a role for chemokines in the initiation of atherosclerosis. Nature 394: 894–897. 9732872 10.1038/29788

[19] Ait-Oufella H, Taleb S, Mallat Z, Tedgui A (2011) Recent advances on the role of cytokines in atherosclerosis. Arteriosclerosis, thrombosis, and vascular biology 31: 969–979. 10.1161/ATVBAHA.110.207415 21508343

[20] Uratsuji H, Tada Y, Kawashima T, Kamata M, Hau CS, et al (2012) P2Y6 receptor signaling pathway mediates inflammatory responses induced by monosodium urate crystals. J Immunol 188: 436–444. 22102722 10.4049/jimmunol.1003746

[21] Linton MF, Atkinson JB, Fazio S (1995) Prevention of atherosclerosis in apolipoprotein E-deficient mice by bone marrow transplantation. Science 267: 1034–1037. 7863332 10.1126/science.7863332

[22] Daugherty A, Cassis L (1999) Chronic angiotensin II infusion promotes atherogenesis in low density lipoprotein receptor −/− mice. Annals of the New York Academy of Sciences 892: 108–118. 10842656 10.1111/j.1749-6632.1999.tb07789.x

[23] Daugherty A, Manning MW, Cassis LA (2000) Angiotensin II promotes atherosclerotic lesions and aneurysms in apolipoprotein E-deficient mice. The Journal of clinical investigation 105: 1605–1612. 10841519 10.1172/JCI7818 PMC300846

[24] Boisvert WA, Santiago R, Curtiss LK, Terkeltaub RA (1998) A leukocyte homologue of the IL-8 receptor CXCR-2 mediates the accumulation of macrophages in atherosclerotic lesions of LDL receptor-deficient mice. The Journal of clinical investigation 101: 353–363. 9435307 10.1172/JCI1195 PMC508574

[25] Gu L, Okada Y, Clinton SK, Gerard C, Sukhova GK, et al (1998) Absence of monocyte chemoattractant protein-1 reduces atherosclerosis in low density lipoprotein receptor-deficient mice. Molecular cell 2: 275–281. 9734366 10.1016/s1097-2765(00)80139-2

[26] Boisvert WA, Curtiss LK (1999) Elimination of macrophage-specific apolipoprotein E reduces diet-induced atherosclerosis in C57BL/6J male mice. Journal of lipid research 40: 806–813. 10224149

[27] Ruiz-Ortega M, Lorenzo O, Ruperez M, Esteban V, Suzuki Y, et al (2001) Role of the renin-angiotensin system in vascular diseases: expanding the field. Hypertension 38: 1382–1387. 11751722 10.1161/hy1201.100589

[28] Rowe VL, Stevens SL, Reddick TT, Freeman MB, Donnell R, et al (2000) Vascular smooth muscle cell apoptosis in aneurysmal, occlusive, and normal human aortas. Journal of vascular surgery 31: 567–576. 10709071

[29] Wang YX, Martin-McNulty B, da Cunha V, Vincelette J, Lu X, et al (2005) Fasudil, a Rho-kinase inhibitor, attenuates angiotensin II-induced abdominal aortic aneurysm in apolipoprotein E-deficient mice by inhibiting apoptosis and proteolysis. Circulation 111: 2219–2226. 15851596 10.1161/01.CIR.0000163544.17221.BE

[30] Hou M, Harden TK, Kuhn CM, Baldetorp B, Lazarowski E, et al (2002) UDP acts as a growth factor for vascular smooth muscle cells by activation of P2Y(6) receptors. Am J Physiol Heart Circ Physiol 282: H784–792. 11788430 10.1152/ajpheart.00997.2000

[31] Schiller NK, Kubo N, Boisvert WA, Curtiss LK (2001) Effect of gamma-irradiation and bone marrow transplantation on atherosclerosis in LDL receptor-deficient mice. Arteriosclerosis, thrombosis, and vascular biology 21: 1674–1680. 10.1161/hq1001.096724 11597944

