# Supplementary figures and images for: P2Y6 Receptor Potentiates Pro-Inflammatory Responses in Macrophages and Exhibits Differential Roles in Atherosclerotic Lesion Development
Source: PLoS One. 2014 Oct 31;9(10):e111385. doi: 10.1371/journal.pone.0111385 (PMC4216081; doi:10.1371/journal.pone.0111385)

# Supplemental Figure S1

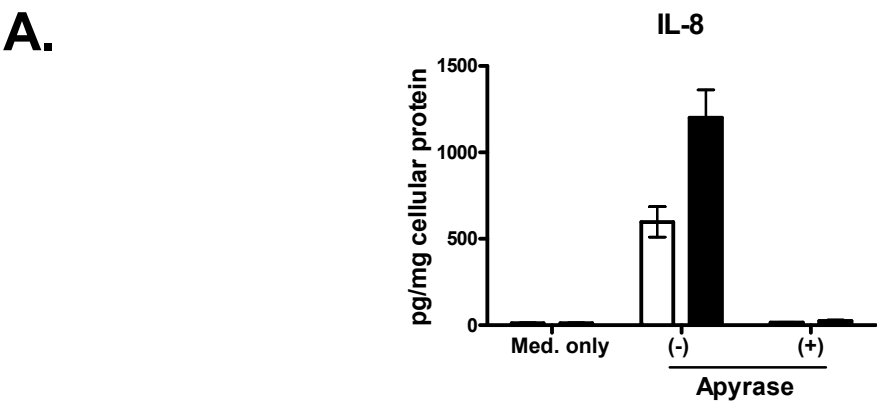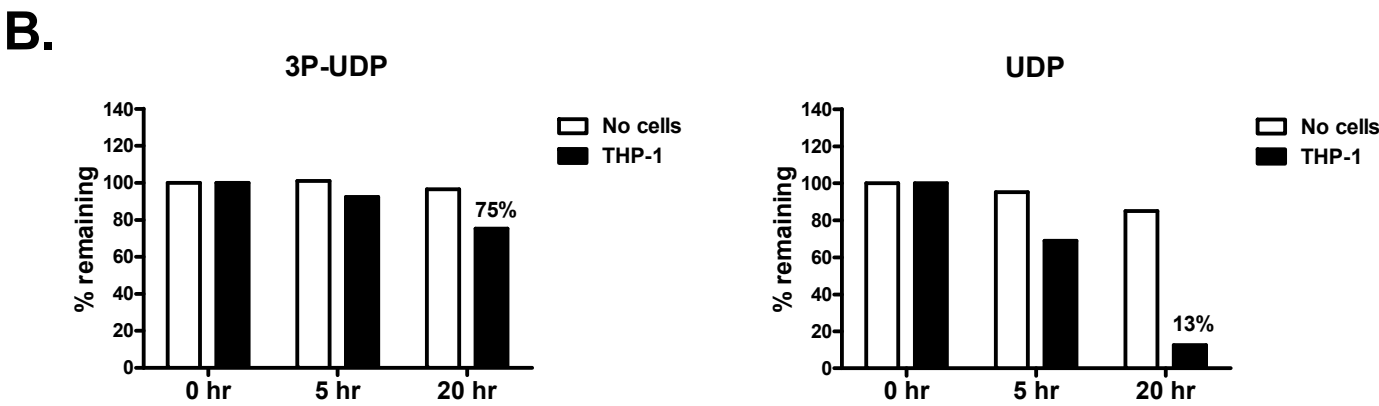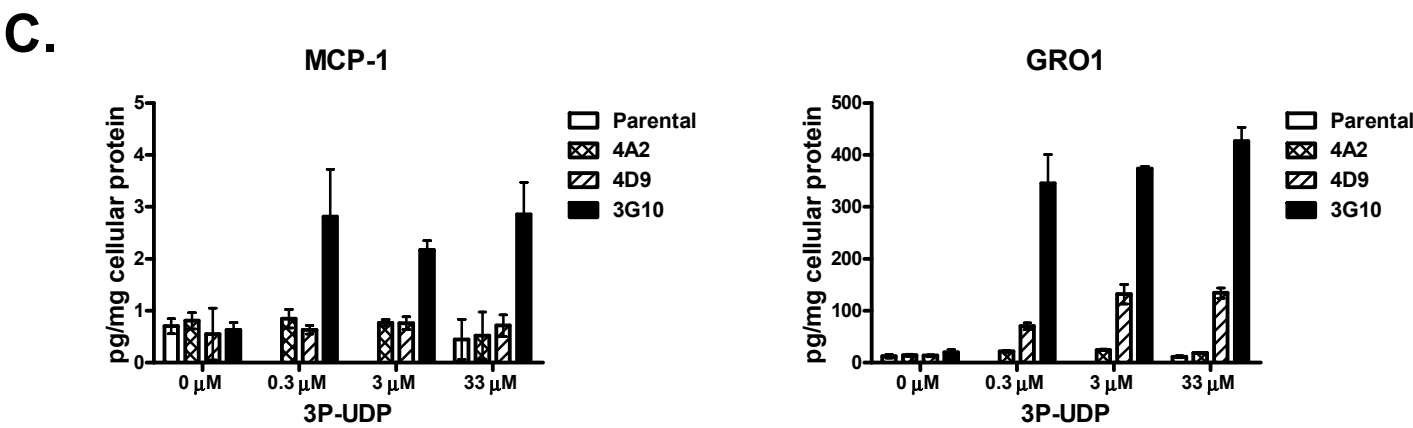

Supplement: Figure S1 — P2Y6 Agonist-Stimulated Release of Cytokines in Cell Lines and Agonist Stability. A) 1321N1_P2Y6 stable cell line (clone 3G10) was incubated with UDP and 3P-UDP (33 µM) for 5 hours in the presence or absence of 1 unit/ml apyrase. After incubation, IL-8 in culture medium was measured by ELISA. B) THP-1 macrophages were incubated with UDP and 3P-UDP (33 µM) for indicated times and the level of UDP and 3P-UDP in culture medium quantified by liquid chromatography and mass spectrometry as described in Materials and Methods. The same experiment was carried out also in the absence of cells. Data were expressed as % remaining relative to 0 hr time point. C) 1321N1 clones were incubated for 20 hrs with increasing concentrations of 3P-UDP and cytokines in cell culture medium quantified using multiplex ELISA as described in Figure 1 and Materials and Methods. Each cytokine was normalized to cellular protein content. The data are presented as mean +/− SD of replicate wells. (PDF) [file pone.0111385.s001.pdf]

# Supplemental Figure S2

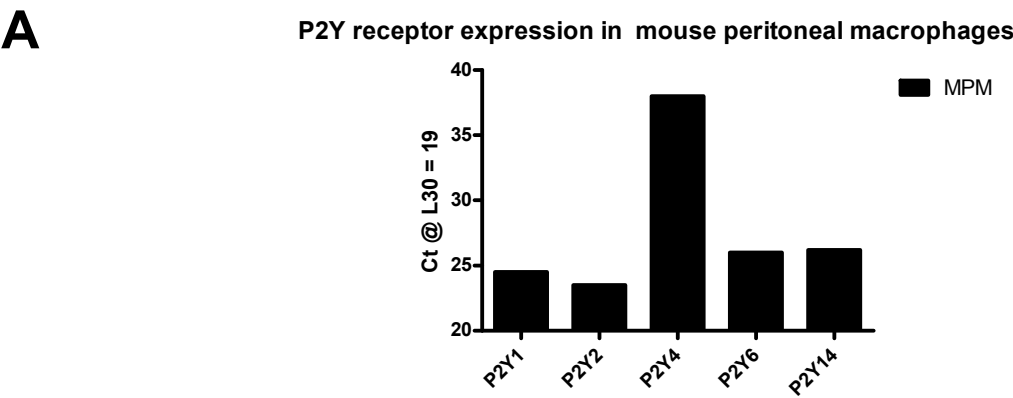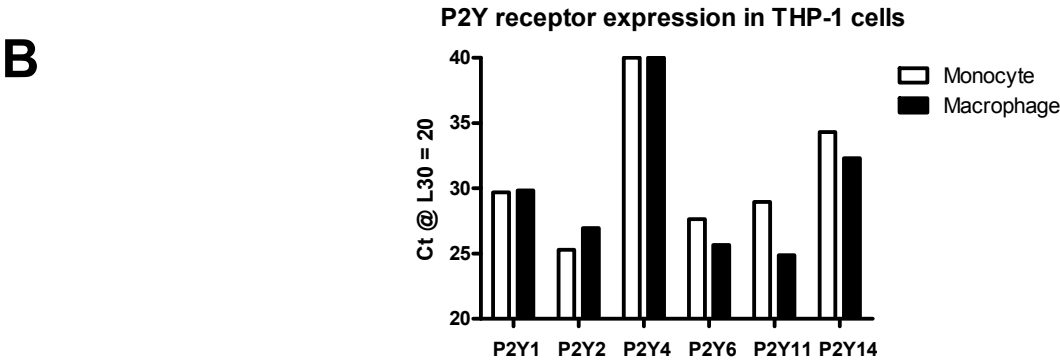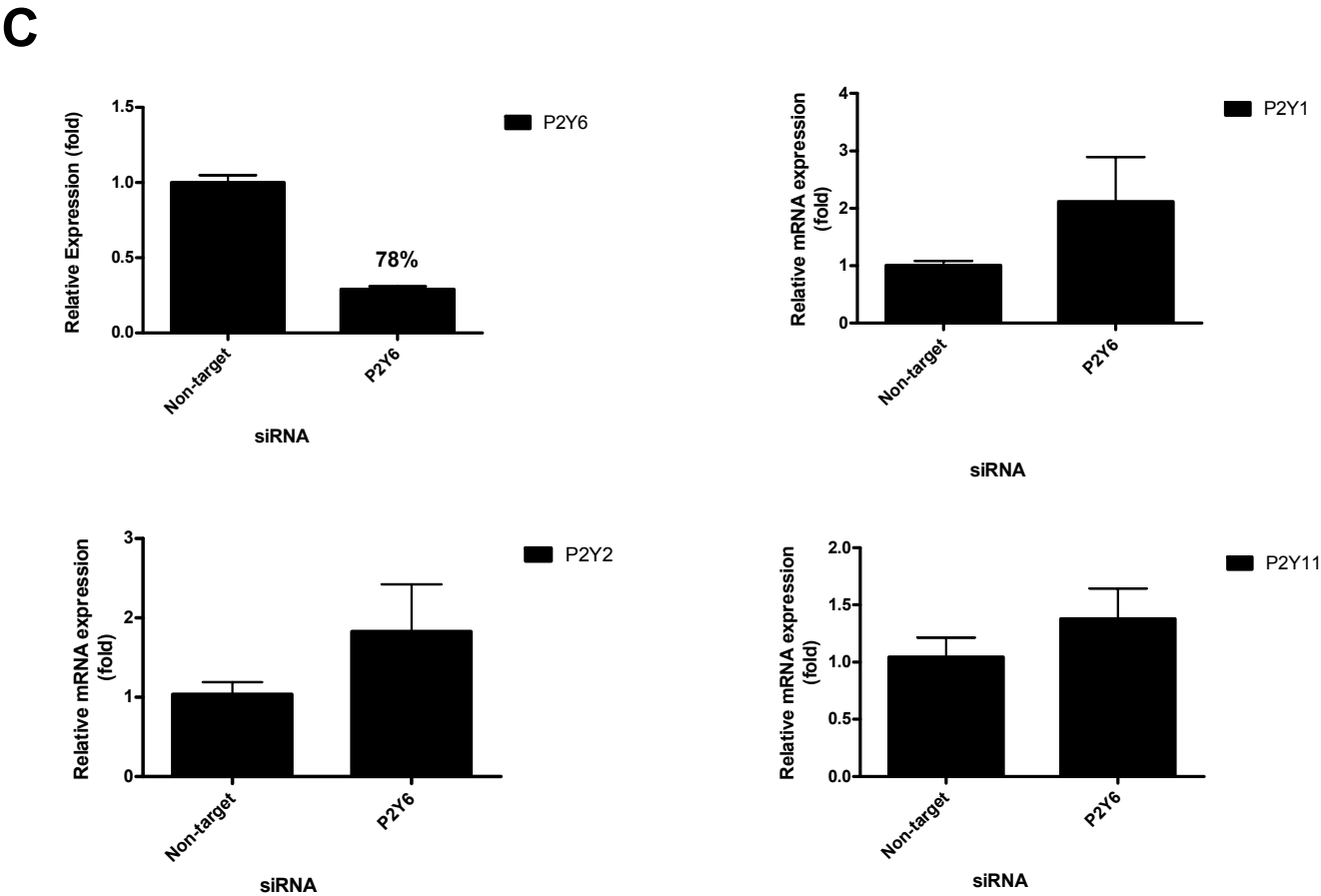

Supplement: Figure S2 — P2Y Receptors in Macrophages. Relative expression of P2Y receptors in mouse primary peritoneal macrophages (A) and THP-1 cells before and after differentiation (B) was analyzed by quantitative PCR. The Threshold Cycle (Ct) value for each P2Y was normalized to RPL30 (set at Ct = 20) and plotted to illustrate relative abundance when compared to RPL30 housekeeping gene (Ct = 20) or genes expressed at the very low levels (Ct> 35). C) P2Y6 siRNA treatment of THP-1 macrophages described in Figure 2A selectively reduced P2Y6 expression level (78% when compared to non-targeted oligonucleotide) and did not lead to reduced expression of other measured P2Y receptors (P2Y1, P2Y2 and P2Y11). (PDF) [file pone.0111385.s002.pdf]

# Supplemental Figure S4

## MSU +/- 3P-UDP

A

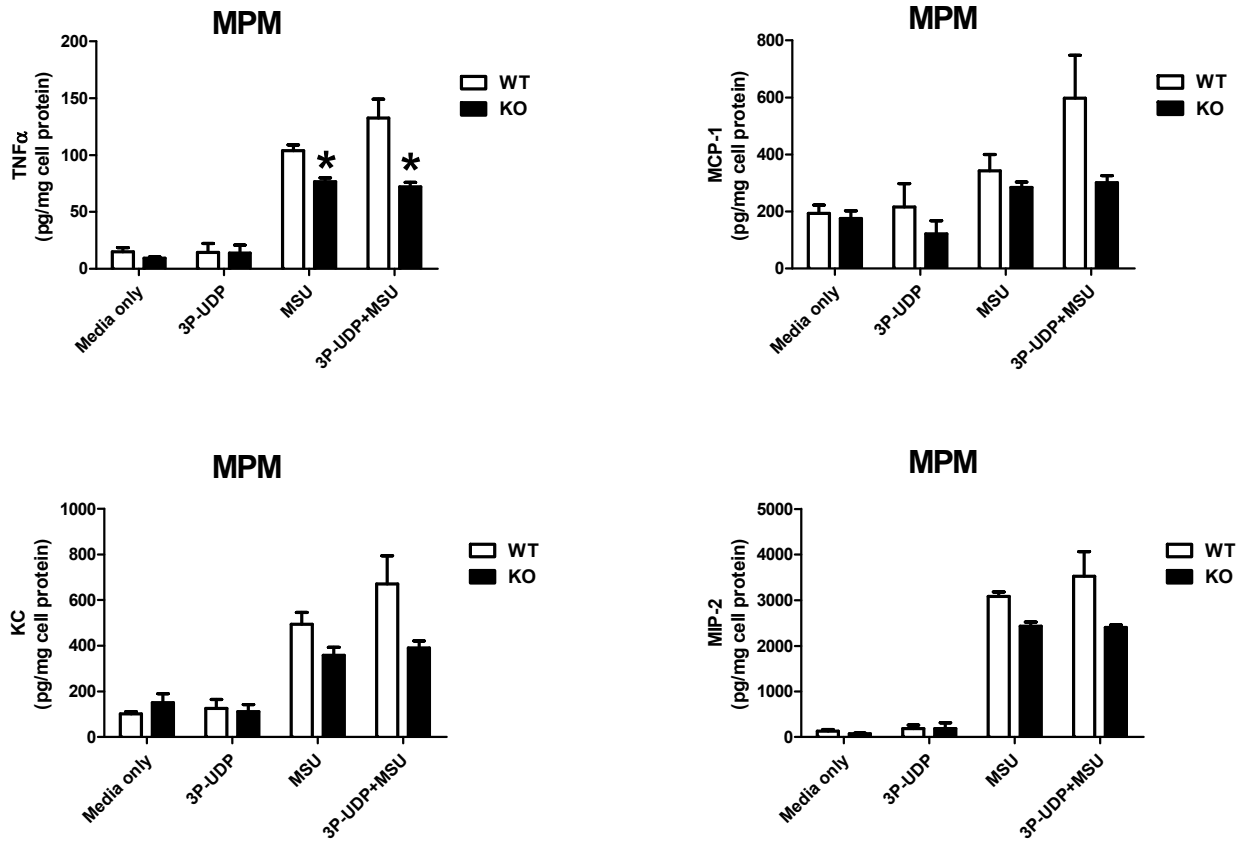

B

## TNFα +/- 3P-UDP

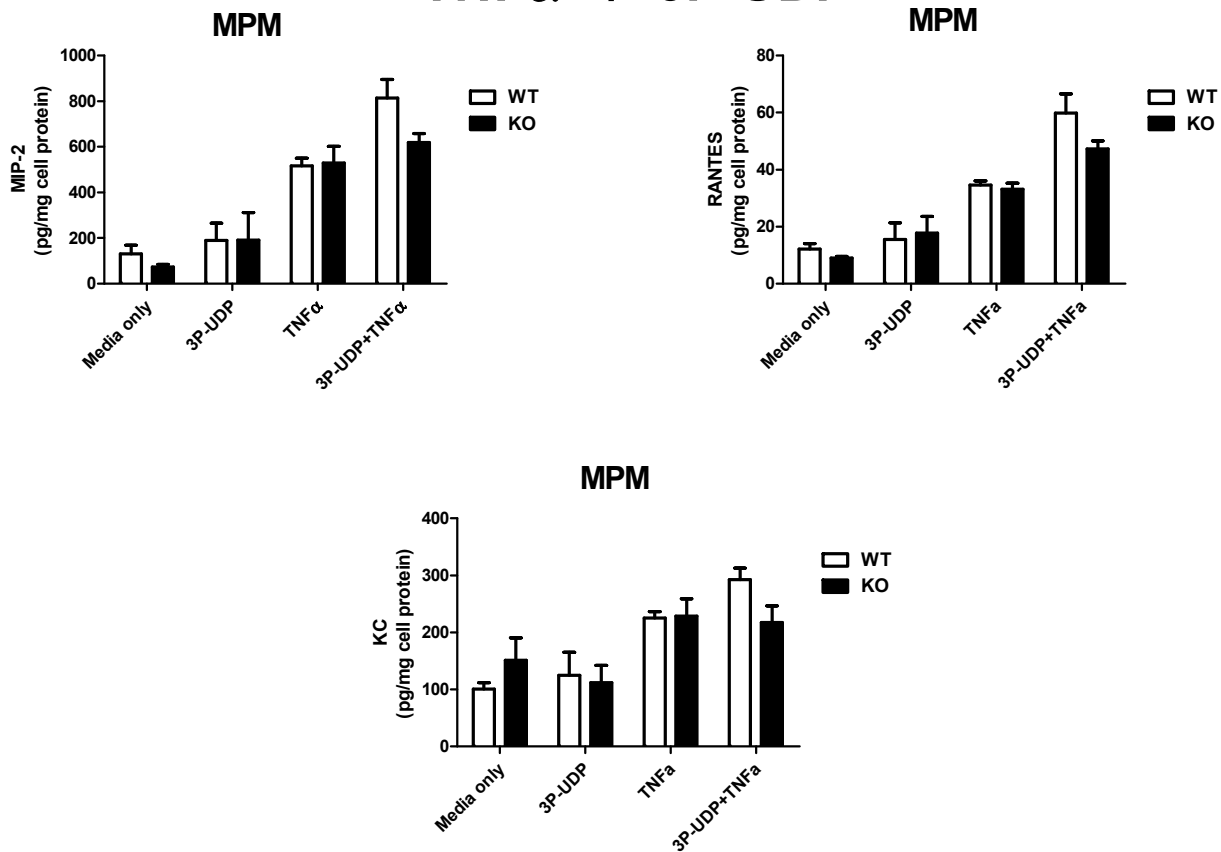

Supplement: Figure S4 — Attenuated Responsiveness of MPMs Isolated from P2Y6 KO Mice Post 3P-UDP and MSU or TNF-α Challenge. Thioglycollate-elicited MPMs were isolated from P2Y6 KO mice and incubated for 16 hrs with A) 3P-UDP (33 µM) +/− MSU (200 µg/ml) or B) 3P-UDP (33 µM) +/− TNF-α (0.1 ng/ml). Cytokines in cell culture medium were analyzed using multiplex ELISA. Results are presented as mean +/− SD from replicate wells. Similar results were obtained in multiple experiments using different animals. Only cytokines which displayed trend or were statistically significantly different between KO and WT are shown. Significance KO vs. WT: *p<0.05. (PDF) [file pone.0111385.s004.pdf]

# Supplemental Figure S5

A.

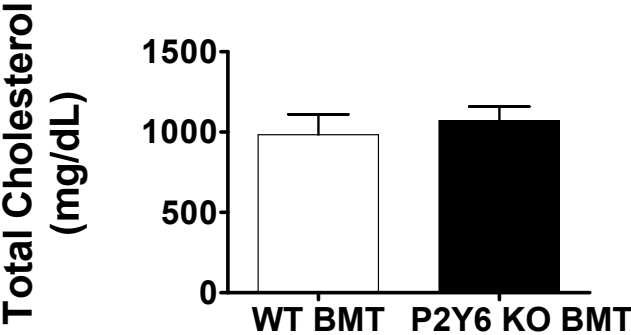

B.

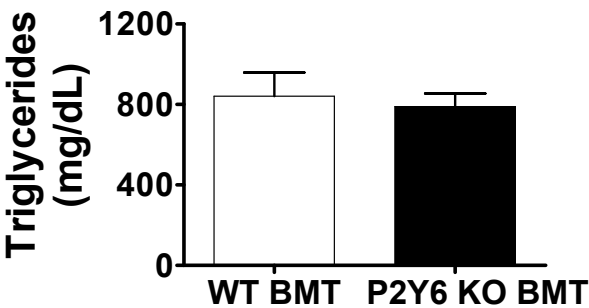

C.

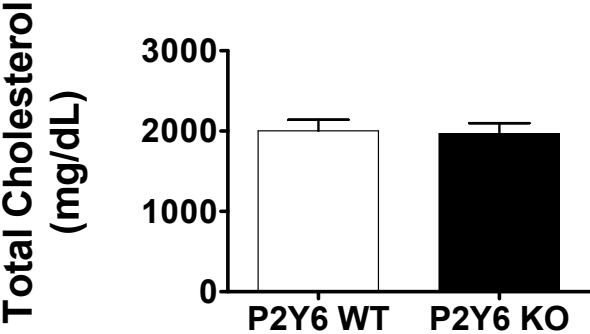

D.

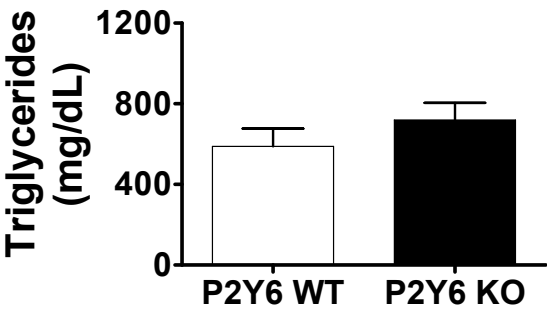

E.

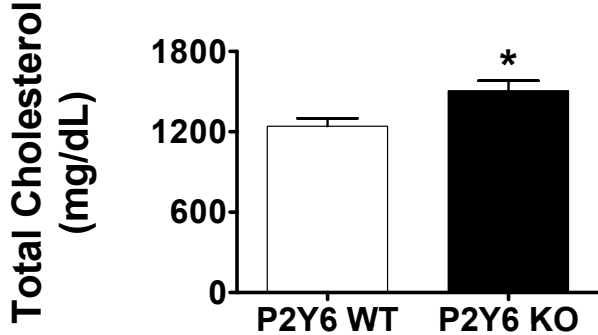

F.

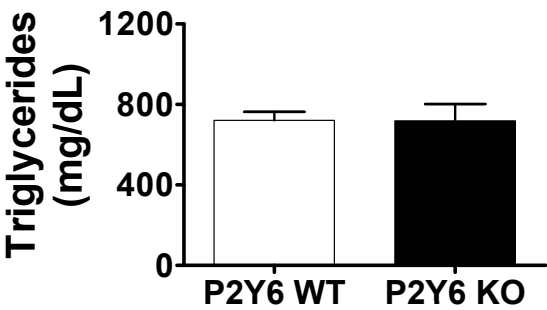

Supplement: Figure S5 — Plasma Cholesterol and Triglycerides. Total plasma cholesterol and triglycerides from P2Y6 WT and P2Y6 KO mice. Bone marrow transplantation study: A) Plasma total cholesterol and B) triglycerides in male mice (n = 9, P2Y6 WT BMT; n = 14, P2Y6 KO BMT). Western diet study: C) Plasma total cholesterol and D) triglycerides in female mice (n = 13, P2Y6 WT; n = 14, P2Y6 KO). Angiotensin II accelerated atherosclerosis study: E) Plasma total cholesterol and F) triglycerides in male mice (n = 14, P2Y6 WT; n = 12, P2Y6 KO). Significance KO vs. WT: *p<0.01. Values are mean ± SE. (PDF) [file pone.0111385.s005.pdf]

# Supplemental Figure S6

A

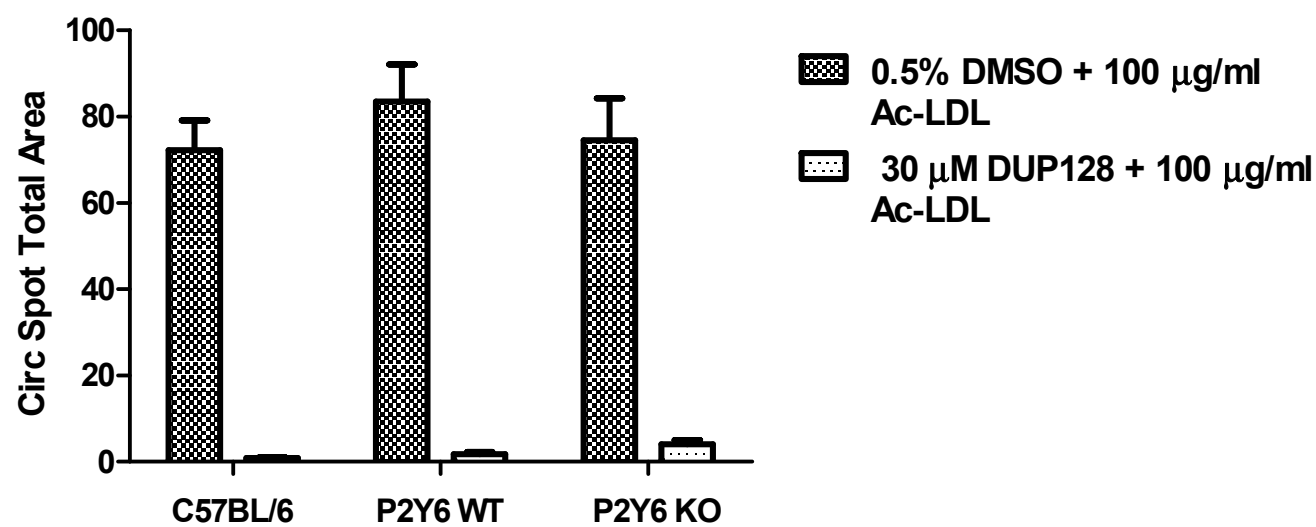

B

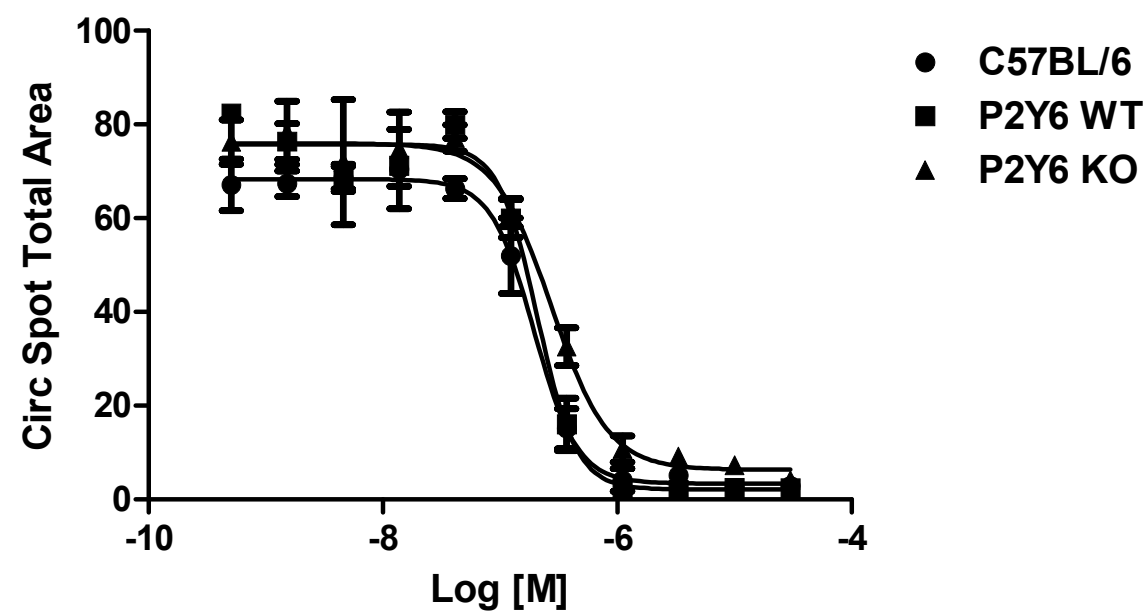

Supplement: Figure S6 — Acetylated LDL Uptake by MPMs. MPMs isolated from P2Y6 KO mice, their WT littermates or age matched C57BL/6 mice were incubated for 20 hrs with 100 µg/ml of acetylated LDL (Biomedical Technologies) in the presence of 0.1% lipid-free BSA and the ACAT inhibitor DUP128 or DMSO. Cells were fixed and stained with Nile Red and Hoechst 33342. Neutral lipid content measured by high-content imaging and quantified by the Compartmental Analysis Bioapplication. Lipid accumulation was expressed as CircSpotTotalArea. The data are presented as mean +/−SD of replicate wells from a representative experiment. A) Quantification of cellular neutral lipid droplet area in DMSO vs. DUP128 (30 µM) treated MPMs from P2Y6 KO, WT and C57BL/6 mice. B) Concentration response curve for DUP128 in MPMs isolated from P2Y6 KO, WT and C57BL/6 mice. (PDF) [file pone.0111385.s006.pdf]
