# Supplementary material for: P2Y6 Receptor Potentiates Pro-Inflammatory Responses in Macrophages and Exhibits Differential Roles in Atherosclerotic Lesion Development
Source: PLoS One. 2014 Oct 31;9(10):e111385. doi: 10.1371/journal.pone.0111385 (PMC4216081; doi:10.1371/journal.pone.0111385)
Supplement: Figure S3 — Phenotypic Characterization of P2Y6 KO Mice and WT Littermate Controls. Immunology and Cardiovascular/Metabolism studies were carried out with mice ranging in age from 12 to 25 weeks. In most cases, male mice were used. Results from studies using female mice are underlined. With few exceptions, most assays did not reveal differences between strains. *Note: the increase in bone density detected in this assay by DEXA scan was not observed in a subsequent cohort of mice by micro computed tomography imaging. (PDF) [file pone.0111385.s003.pdf]

Supplemental Figure S3

Immunology

	RESULTS
OVA-Induced Allergic Pulmonary Inflammation	↑ & ↓ inflammatory cells
LPS-Induced Pulmonary Inflammation	No differences
SRBC-Delayed Type Hypersensitivity (DTH)	No differences
Thioglycollate-Induced Monocyte Infiltration	No differences
Ex Vivo Cytokine Production by Monocytes Stimulated with LPS	No differences
Hematological Analysis of Peripheral Blood	No differences
FACS Analysis of Splenocytes	No differences
Cytokine Production by CD4+ Cells	No differences
B and CD4+ T Cell Proliferation *In Vitro*	No differences
Dextran Sulfate Sodium (DSS)-induced Colitis	No differences
In Vivo LPS Challenge Without D-Galactosamine	↓ IL-1β & TNFα

Cardiovascular and Metabolism

Blood Pressure and Heart Rate in the N-methyl-L-arginine (L-NMA) Induced Hypertension Model	No differences
The Response to the High-Fat Diet Challenge	No differences
Individual Assays Included in the High Fat Diet Challenge	No differences
Body Weight Measurements	No differences
Body Composition by Dual-Energy X-ray Absorptiometry (DEXA)	No differences
Oral Glucose Tolerance Test (OGTT)	No differences
Body Composition by Dual-Energy X-ray Absorptiometry (DEXA) Females	No differences
Excised Bone DEXA	↑ bone density*
Tail Bleeding Time	No differences
